# Prefusion spike protein conformational changes are slower in SARS-CoV-2 than in SARS-CoV-1

**DOI:** 10.1016/j.jbc.2022.101814

**Published:** 2022-03-10

**Authors:** Vivek Govind Kumar, Dylan S. Ogden, Ugochi H. Isu, Adithya Polasa, James Losey, Mahmoud Moradi

**Affiliations:** Department of Chemistry and Biochemistry, University of Arkansas, Fayetteville, Arkansas, USA

**Keywords:** SARS-CoV, SARS-CoV-2, coronavirus disease 2019, molecular dynamics, conformational change, ACE2, angiotensin-converting enzyme 2, CoV, coronavirus, DNA, dynamic network analysis, MD, molecular dynamics, NTD, N-terminal domain, PC, principal component, PCA, principal component analysis, PDB, Protein Data Bank, RBD, receptor-binding domain, RBM, receptor-binding motif, SARS, severe acute respiratory syndrome, SARS-CoV, severe acute respiratory syndrome coronavirus, SMD, steered molecular dynamics, smFRET, single-molecule FRET, SPR, surface plasmon resonance

## Abstract

Within the last 2 decades, severe acute respiratory syndrome coronaviruses 1 and 2 (SARS-CoV-1 and SARS-CoV-2) have caused two major outbreaks; yet, for reasons not fully understood, the coronavirus disease 2019 pandemic caused by SARS-CoV-2 has been significantly more widespread than the 2003 SARS epidemic caused by SARS-CoV-1, despite striking similarities between these two viruses. The SARS-CoV-1 and SARS-CoV-2 spike proteins, both of which bind to host cell angiotensin-converting enzyme 2, have been implied to be a potential source of their differential transmissibility. However, the mechanistic details of prefusion spike protein binding to angiotensin-converting enzyme 2 remain elusive at the molecular level. Here, we performed an extensive set of equilibrium and nonequilibrium microsecond-level all-atom molecular dynamics simulations of SARS-CoV-1 and SARS-CoV-2 prefusion spike proteins to determine their differential dynamic behavior. Our results indicate that the active form of the SARS-CoV-2 spike protein is more stable than that of SARS-CoV-1 and the energy barrier associated with the activation is higher in SARS-CoV-2. These results suggest that not only the receptor-binding domain but also other domains such as the N-terminal domain could play a crucial role in the differential binding behavior of SARS-CoV-1 and SARS-CoV-2 spike proteins.

Within the last 2 decades, severe acute respiratory syndrome (SARS) coronavirus 1 (SARS-CoV-1) and SARS coronavirus 2 (SARS-CoV-2) (1, 2, 3) (4, 5, 6, 7, 8) have caused the SARS epidemic and coronavirus disease 2019 pandemic, respectively. Various studies have shown that CoV-2 is more easily transmissible between humans in comparison to CoV-1 (9, 10, 11, 12). However, given the striking similarity of the two viruses, the molecular-level explanation of their differential transmissibility is largely missing. The two viruses share several highly conserved structural and functional features (4, 13, 14). The spike protein plays a crucial role in the infection process (13, 15, 16, 17) and has been the primary target of various candidate drugs and vaccines (18, 19, 20, 21, 22, 23, 24, 25, 26).

CoV-1 and CoV-2 spike proteins have a high sequence identity of approximately 79% (4), and the receptor-binding domains (RBDs) of both proteins interact with the human angiotensin-converting enzyme 2 (ACE2) receptor (9, 16, 17, 27, 28, 29, 30, 31). Studies have shown that several regions of the CoV-2 spike protein are susceptible to mutations, with the RBD being particularly vulnerable in this regard (32, 33, 34, 35). It is possible that therapeutic agents targeting only the RBD–ACE2 interaction might eventually be rendered ineffective because of the appearance of emerging variants. Therefore, diversifying the hot spots of the protein being targeted by therapeutics and vaccines is essential in increasing their long-term efficacy. The current study provides a rational framework for such directions by systematically studying the differential behavior of the CoV-1 and CoV-2 spike proteins, highlighting significant regions of the protein that are involved in the activation process, that is, a large-scale conformational change in the prefusion spike protein, which occurs prior to ACE2 binding.

Recently, several cryo-EM and computational studies have shed light on the differential receptor-binding behavior of the CoV-1 and CoV-2 spike proteins (9, 17, 27, 36, 37). The RBD of the spike protein undergoes a large-scale conformational transition from an inactive “down” position to an active “up” position in order to access the ACE2 receptors on the host–cell surface (9, 17, 27, 38, 39, 40). Experimental studies investigating the binding affinity of the spike protein RBD for the ACE2-peptidase domain have produced varying results. Using surface plasmon resonance (SPR) and flow cytometry techniques, respectively, Wrapp *et al.* (27) and Tai *et al*. (17) have reported that the CoV-2 RBD has a higher binding affinity for ACE2-peptidase domain than the CoV-1 RBD. For instance, the SPR-based assay shows that the dissociation constant of the CoV-2 spike protein (*K*_*d*_ ≈ 14.7 nM) is 10 to 20 times lower than that of the CoV-1 spike protein (27, 41). In a different study, biolayer interferometry has shown that the CoV-2 dissociation constant (*K*_*d*_ ≈ 1.2 nM) is only four times lower than that of CoV-1, indicating that the binding affinities are generally comparable (9). Such quantitative inconsistencies emphasize the need to improve our understanding of the mechanistic aspects of the RBD–ACE2 interaction. A disadvantage of experimental techniques like SPR and biolayer interferometry is that they require the protein to be immobilized prior to measuring the binding affinity (42, 43). This introduces a level of bias into these experimental assays, particularly if the binding behavior of a protein is conformation dependent, as is the case for the coronavirus spike proteins. One may argue that some studies have neglected the fact that the binding process involves not only the RBD–ACE2 interaction but also the spike protein activation, a large-scale conformational change with a potentially significant contribution to the differential binding behavior of SARS-CoV-1 and SARS-CoV-2. Therefore, to gain a clearer understanding of the enhanced infectivity of SARS-CoV-2, “effective binding” involving both the RBD–ACE2 interaction and the spike protein activation/inactivation process needs to be investigated. Here, we focus on the latter, which has received less attention in the literature.

Cryo-EM studies have successfully resolved structures of both spike proteins in the inactive state, active unbound state, and active ACE2-bound state (9, 27, 31, 38, 44). However, cryo-EM and X-ray crystallography studies essentially capture static pictures of specific protein conformations (45, 46, 47). In addition, given the substantial differences in the experimental and physiological conditions, it is not clear whether all relevant conformational states are captured using these techniques. For instance, a recent single-molecule FRET (smFRET) study has captured an alternative inactive conformation for the CoV-2 spike protein (48) that is not consistent with those obtained from cryo-EM. It is thus important to investigate the differential conformational landscapes of the CoV-1 and CoV-2 spike proteins in terms of both important functional states and their dynamics. For this purpose, we use an extensive set of microsecond-level unbiased and biased molecular dynamics (MD) simulations. Here, we make certain assumptions to be able to make progress toward deciphering the differential behavior of the two spike proteins, such as relying on cryo-EM structures as our initial models, excluding the unresolved transmembrane domain of the spike protein, and excluding the glycan chains in the simulations. However, we treat the spike proteins of both viruses similarly so that a reliable comparison can be made.

Allowing for the fact that this study has certain limitations as discussed previously, our extensive all-atom equilibrium MD simulations show that the active CoV-2 spike protein is potentially more stable than the active CoV-1 spike protein. We also report that the RBD of the active CoV-1 spike protein can undergo a spontaneous conformational transition to a pseudoinactive state characterized by the interaction of the N-terminal domain (NTD) and RBD, a state not observed in any of the previous experimentally reported structures of the CoV-1 or CoV-2 spike proteins. This observation is broadly in line with the recent smFRET experimental results indicating the presence of alternative inactive spike protein conformations (48). More specifically, electrostatic interaction analyses reveal that unique salt-bridge interactions between the NTD and RBD of the CoV-1 spike protein are involved in the major conformational transition observed in our simulations. No large-scale conformational changes occur in any of the active CoV-2 spike protein simulations or any of the inactive CoV-1 or CoV-2 spike protein simulations within the timescale of our unbiased MD simulations (5 μs).

In order to investigate the longer timescale conformational dynamics inaccessible to unbiased simulations (49), we have also employed extensive steered molecular dynamics (SMD) simulations (50) along with nonequilibrium work calculations (51) to make a semiquantitative comparison between the two proteins (52, 53). The SMD simulations shed light on the energetics of the conformational change associated with the activation and inactivation processes. The results obtained from these enhanced simulations strongly suggest that the energy barriers for such conformational transitions are significantly lower for the CoV-1 spike protein and that conformational changes occur more slowly for the CoV-2 spike protein. This provides an explanation for the conformational plasticity displayed by the active CoV-1 spike protein in our simulations as well as the relative conformational stability of the active CoV-2 spike protein. The results from our equilibrium and nonequilibrium simulations thus provide a self-consistent picture of the long timescale conformational dynamics of the CoV-1 and CoV-2 spike proteins. We note that our results are not conclusive with regard to the thermodynamics of activation and inactivation. Instead, they provide a semiquantitative picture of the kinetics. The propensity of the active CoV-2 spike protein to maintain the “up” RBD conformation for a longer period as compared with CoV-1 might explain why the CoV-2 has a better chance of remaining bound to ACE2, long enough to allow for the next step in the viral entry process, which in turn could potentially be linked to the comparatively high human-to-human transmissibility of CoV-2.

## Results

We have performed 5-μs-long unbiased all-atom MD simulations of both inactive and active CoV-1 and CoV-2 spike proteins in explicit water. The active CoV-1 and CoV-2 simulations were repeated additionally twice for another 5 μs each (see Supporting information MD simulation details). We have also performed 80 independent nonequilibrium SMD simulations of the CoV-1 and CoV-2 spike proteins, each for 100 ns, to compare the activation and inactivation of CoV-1 and CoV-2 spike proteins that are otherwise generally inaccessible to unbiased MD. We have thus generated 40 μs of equilibrium and 8 μs of nonequilibrium simulation trajectories in aggregate.

**Figure 1:**
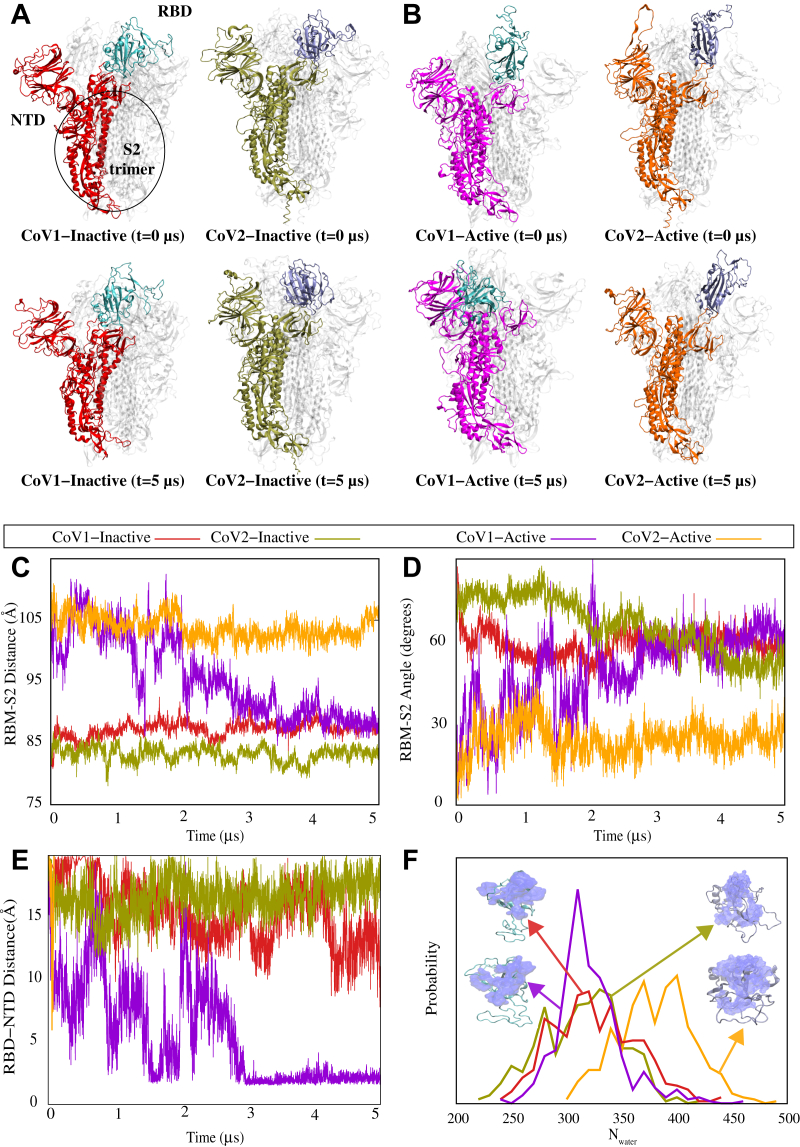
**Unbiased simulations of the CoV-1 and CoV-2 spike proteins show a differential dynamic behavior.***A* and *B*, the initial and final MD snapshots of CoV-1 and CoV-2 spike proteins starting from both inactive and active states. Protomer A in each protein is *colored*, and protomers B and C are shown in *white*. The RBD of the colored protomer has a distinctive color from the rest of the protomer. Based on multiple repeats of these simulations, we have observed that the active form of the CoV-2 spike protein is consistently more stable than the active CoV-1 spike protein. The active CoV-1 spike protein transitions spontaneously to a pseudoinactive conformation. *C*, the center-of-mass distance between the S2 trimer of the spike protein and the RBM of protomer A shown as a function of time. *D*, the angle between the S2 trimer of the spike protein and RBM of protomer A shown as a function of time. *E*, minimum distance between the NTD and RBD of protomer A as a function of time for CoV-1 and CoV-2 spike proteins in both active and inactive state simulations. *F*, probability density map of water within 5 Å of the RBM for the final 500 ns of simulation. *C*–*F*, the same color code is used to represent CoV-1-inactive (*red*), CoV-1-active (*magenta*), CoV-2-inactive (*olive-green*), and CoV-2-active (*orange*). CoV, coronavirus; MD, molecular dynamics; NTD, N-terminal domain; RBD, receptor-binding domain; RBM, receptor-binding motif.

Within the timescale of our unbiased equilibrium simulations (*i.e.*, 5 μs), the inactive forms of both CoV-1 and CoV-2 spike proteins do not undergo any major conformational transitions, with the RBDs remaining in the “down” position (Fig. 1*A*) (9, 38). On the other hand, a spontaneous large-scale conformational change occurs in the active CoV-1 spike protein simulation (Fig. 1*B*), with the RBD moving from an active “up” position to a pseudoinactive “down” conformation that is different from the inactive conformation in the cryo-EM structure (38). This spontaneous conformational transition appears to occur because of interactions between the NTD and RBD of the CoV-1 spike protein (Fig. 1*B*). Unlike the active CoV-1, the active CoV-2 spike protein does not undergo any large-scale conformational transitions and remains in the active state within the simulations of 5 μs (Fig. 1*B*). Movie S1 demonstrates the differential behavior of CoV-1 and CoV-2 clearly.

To examine the reproducibility of the aforementioned observations, the active CoV-1 and CoV-2 simulations were repeated twice (see Supporting information MD simulation details). Consistent with set 1, the active CoV-2 simulations do not show any significant conformational change in sets 2 and 3. The active CoV-1 simulations, on the other hand, undergo some significant conformational change in set 2 and set 3; although these conformational changes are not the same in the three different repeats. The dramatic change from the “up” to “down” (or pseudoinactive) conformation of the CoV-1 spike protein is only observed in set 1; however, all three sets show some significant conformational changes that are not observed in any of the CoV-2 simulations. RMSD (Fig. S1) and root mean square fluctuation (Fig. S2) analyses demonstrate the relative stability of the active CoV-2 as compared with the active CoV-1 spike protein. A comparison of individual protomer RMSDs from all three repeats of the active CoV-1 and CoV-2 spike protein trajectories clearly shows that the active CoV-1 spike protein is less stable overall as compared with the active CoV-2 (Fig. S1). Similarly, root mean square fluctuation analysis indicates that the RBD and NTD regions of the active CoV-1 spike protein fluctuate more than the corresponding regions of the active CoV-2 (Fig. S2).

In order to quantify the spontaneous conformational transition that occurs in the active CoV-1 spike protein, we measured the center-of-mass distance between the receptor-binding motif (RBM) of protomer A and the S2 trimer of the spike protein (Fig. 1*C*). The RBM–S2 distance remains stable for both inactive states at ≈85 Å over 5 μs. For both the CoV-1 and CoV-2 active states, the RBM–S2 distance is initially ≈100 Å but decreases to ≈85 Å for CoV-1 after 2 μs (Fig. 1*C*). This analysis clearly demonstrates that the final conformation adopted by the RBD of the active CoV-1 spike protein is similar to the inactive state RBD conformations of both CoV-1 and CoV-2, in terms of the RBM–S2 trimer distance (Fig. 1*C*). On the other hand, the RBM–S2 trimer distance for the active CoV-2 spike protein remains relatively unchanged over 5 μs (Fig. 1*C*), consistent with the molecular images shown in Figure 1, *A* and *B*. Similarly, the angle between the RBM of protomer A and the S2 trimer remains relatively unchanged for the CoV-2 active state, whereas the CoV-1 active simulation shows a behavior during the last 3 μs that is similar to that of the inactive states of CoV-1 and CoV-2 (Fig. 1*D*).

The RBD–NTD contact analysis also demonstrates the RBD–NTD association in the so-called pseudoinactive conformation observed in our CoV-1 simulations. We specifically calculated the minimum distance between the RBD and NTD of protomer A for each system (Fig. 1*E*). While the RBM–S2 distance and angle calculations indicate that the behavior of the CoV-1 active state eventually resembles that of both inactive systems (Fig. 1, *C* and *D*), the NTD–RBD distance calculation showcases the unique behavior of the pseudoinactive CoV-1 spike protein. The NTD–RBD distance of the active protomer in CoV-1 fluctuates considerably over the first 2 μs of the trajectory, after which it decreases sharply to settle down around 2 Å (Fig. 1*E*). This clearly demonstrates that the RBD of the pseudoinactive CoV-1 spike protein, that results from the inactivation of the active CoV-1 spike, is in close proximity to the NTD as also shown in the cartoon representations (Fig. 1*B*). This is not observed during any of the simulations of active CoV-2 spike protein or either of the inactive spike proteins (Fig. 1, *A*, *B*,and *E*), thus indicating that the pseudoinactive conformation adopted by the initially active CoV-1 spike protein is unique.

The RBM hydration analysis provides more evidence that the pseudoinactive CoV-1 is truly inactive since its exposure to water (as a proxy to ACE2 accessibility) is quite similar to that of inactive CoV-1 and CoV-2 states. This is quantified using the estimated probability distribution for the number of water molecules near the RBM during the last 500 ns of simulations (Fig. 1*F*). The water molecule count for the pseudoinactive state (here, represented by the last 500 ns of the simulation starting with the CoV-1 active state) is considerably lower than that of the CoV-2 active state and is comparable to the counts for the CoV-1/CoV-2 inactive states, further confirming that the active CoV-1 spike protein undergoes a large-scale conformational transition and becomes inactive (Fig. 1*F*).

While the measures discussed previously provide clear evidence that the CoV-2 spike protein behaves more as a stable structure in its active state as compared with the CoV-1 spike protein, more insight can be gained from more systematic analysis techniques such as principal component analysis (PCA) (54) and dynamic network analysis (DNA) (55). For instance, considering the (PC1, PC2) space shows that the region sampled by the active protomer of the CoV-1 spike protein is considerably larger than the region sampled by the corresponding protomers of the CoV-2 spike protein (Fig. S3). Interestingly, the PCA reveals that the most pronounced conformational change (*i.e.*, PC1) is related to the motion of the RBD toward the NTD in the CoV-1 spike protein (Fig. S3). For more PCA-based analysis, see supporting discussion and Figs. S3–S5 in the Supporting information. Similarly, the DNA analysis provides more details on the differential behavior of the spike proteins of CoV-1 and CoV-2. For instance, CoV-1 protomer A (*i.e.*, the active protomer) shows several high interdomain correlations (indicating concerted motions), whereas these correlations are missing in the same protomer of CoV-2 (Fig. S6). Similar trends were observed in all three sets of CoV-1 and CoV-2 active-state simulations (Figs. S7–S10). Interprotomer correlations also highlight the differential behavior of the active CoV-1 and CoV-2 spike proteins (Fig. S11). For more DNA-based analysis, see supporting discussion and Figs. S6–S11 in the Supporting information.

**Figure 2:**
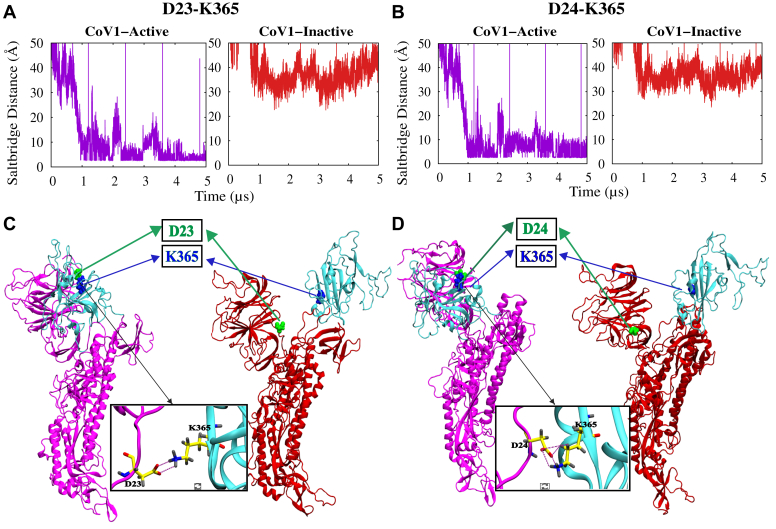
**Unique salt-bridge interactions between the RBD and NTD of the active CoV-1 spike protomer facilitate the transition to a pseudoinactive conformation.***A* and *B*, time series of D23/D24–K365 (*A*/*B*) salt-bridge distances in CoV-1 spike protein simulations. *C* and *D*, visual representations of salt-bridge formation in the initially active CoV-1 protomer A. D23 and D24 (*green*) of the NTD form a salt bridge with K365 (*blue*) of the RBD only in the pseudoinactive state of CoV-1. D23 and D24 are not present in the CoV-2 spike protein. CoV, coronavirus; NTD, N-terminal domain; RBD, receptor-binding domain.

**Figure 3:**
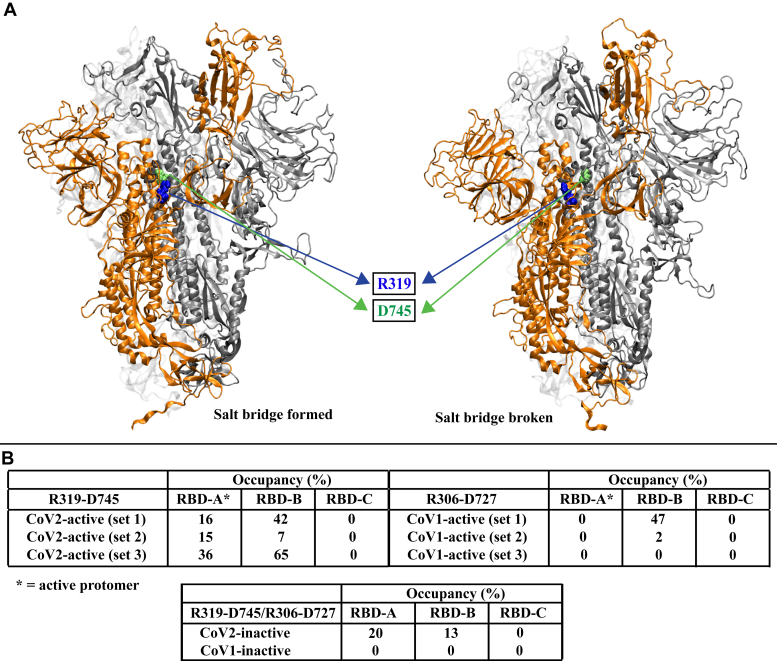
**A conserved residue pair forms a weak salt bridge between the RBD of the active protomer and the S2 region in the CoV-2 spike protein but not in the CoV-1 spike protein.***A*, visual representations of salt-bridge formation and breakage in the active CoV-2 spike protein. R319 (*blue*) in the RBD of the active protomer (*orange*) forms a salt bridge with D745 (*green*) in the S2 region of an adjacent inactive protomer (*silver*). The table (*B*) shows the occupancy (percent) of the R319–D745/R306–D727 salt-bridge interactions for all protomers from all simulation sets. CoV, coronavirus; RBD, receptor-binding domain.

Our extensive electrostatic interaction analysis reveals that the driving force behind the unique conformational transition observed in the initially active CoV-1 spike protein simulation (Fig. 1) is at least partly a set of salt-bridge interactions that are unique to CoV-1. Residues D23 and D24 in the NTD interact with K365 in the RBD, forming stable salt bridges in the active CoV-1 spike protein but not in the inactive state (Fig. 2). These fairly stable salt bridges form around the 1 μs mark (Fig. 2, *A* and *B*), prior to the final movement of the RBD toward the NTD (Fig. 1*E*). Residues D23 and D24 are not conserved in the SARS-CoV-2 spike protein. Differential behavior is also observed for two sets of residues that are conserved in both CoV-1 and CoV-2 spike proteins (Fig. S12). R328 and D578 form a stable salt bridge in both active and inactive CoV-2 spike proteins, whereas R315 and D564 do not form a salt bridge in the CoV-1 spike proteins (Fig. S12*A*). Similarly, R273 and D290 form a stable salt bridge in both active and inactive CoV-2 spike proteins, whereas K258 and D277 do not form a salt bridge in the CoV-1 spike proteins (Fig. S12*B*). In addition, a conserved pair of residues forms an intra-RBD hydrogen bond in the active/inactive CoV-2 spike protein (Y396–E516) and the inactive CoV-1 spike protein (Y383–E502) but not in the active CoV-1 spike protein (Y383–E502) (Fig. S13). An investigation of interactions between the RBD and S2 region reveals that a conserved residue pair (R319 and D745) always forms a weak salt bridge involving the RBD of the active CoV-2 spike protomer, whereas the equivalent residues in the CoV-1 spike protein (R306 and D727) never form a salt bridge involving the active protomer (Fig. 3, *A* and *B*). On the other hand, the RBDs of the inactive protomers are sometimes involved in the formation of this salt bridge in both the CoV-1 and CoV-2 spike proteins (Fig. 3*B*). These electrostatic interactions thus potentially contribute to the relative stability of the active SARS-CoV-2 spike protein.

**Figure 4:**
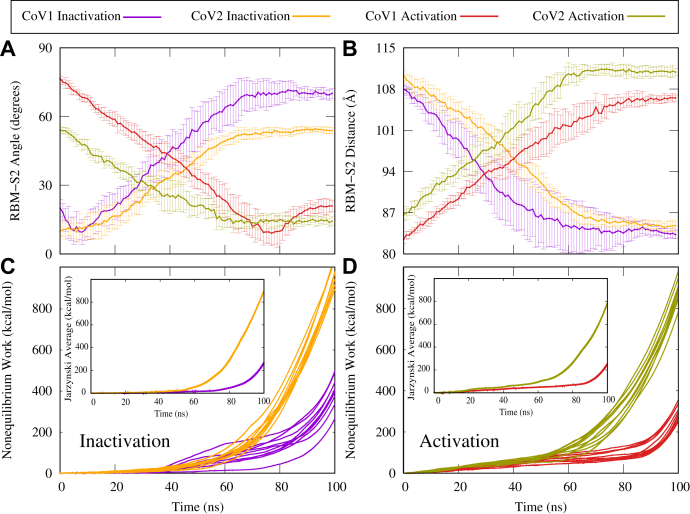
**SMD simulations show that the CoV-2 spike protein has higher energy barriers between active and inactive states as compared with the CoV-1 spike protein.***A*, RBM–S2 angle between the beta-sheet region of the RBM and the alpha-helical region of S2, shown as a function of time during SMD simulations. Protomer activation is characterized by a decrease in the RBM–S2 angle. *B*, RBM–S2 COM distance between the beta-sheet region of the RBM and the alpha-helical region of S2, as shown as a function of time during SMD simulations. Protomer activation is characterized by an increase in the RBM–S2 distance. *C* and *D*, accumulated nonequilibrium work as a function of time during SMD simulations for individual simulations. *Inset*, the Jarzynski average over 10 individual work profiles shown in (*C*) and (*D*). CoV, coronavirus; RBM, receptor-binding motif; SMD, steered molecular dynamics.

SMD simulations were performed to semiquantitatively characterize the energetics of the activation–inactivation process for the CoV-1 and CoV-2 spike proteins. To induce the activation or inactivation of individual protomers, we used the Cα RMSD of each protomer with respect to a target structure (the inactive state for the inactivation process and the active state for the activation process). Ten sets of 100 ns SMD simulations were performed for each system. The conformational transition of an inactive RBD to the active “up” position was accompanied by a decrease in the RBM–S2 angle and an increase in the RBM–S2 distance, as expected (Fig. 4, *A* and *B*). Similarly, the inactivation of an active protomer was characterized by an increase in the RBM–S2 angle and a decrease in the RBM–S2 distance, as expected (Fig. 4, *A* and *B*).

Without performing strict free-energy calculations, we have used nonequilibrium work measurements to compare the energetics of the CoV-1/CoV-2 spike protein activation–inactivation process in a semiquantitative manner. We have previously used similar methods to investigate conformational transitions of other biomolecular systems (52, 53, 56, 57). The accumulated nonequilibrium work measured during the inactivation of an initially active CoV-2 protomer or the activation of an initially inactive CoV-2 protomer is significantly larger than the work measured during the inactivation or activation of the corresponding CoV-1 protomer (Fig. 4, *C* and *D*). Similarly, the change in the associated Jarzynski average is also much higher for the CoV-2 protomers (Fig. 4, *C* and *D*, *inset*). We note that the Jarzynski average would only quantify the true free energy if converged, which requires many more repeats. However, here we are only interested in relative behavior of the CoV-1 and CoV-2 in a qualitative or a semiquantitative manner rather than accurately calculating any free energies (52, 56, 57). These results suggest that the CoV-2 spike protein has slower kinetics, because of higher barriers, in both directions. In other words, the conformational changes associated with activation or inactivation of the spike protein proceeds more slowly in CoV-2 relative to CoV-1. This is in good agreement with our observations on the relative conformational stability of the active CoV-2 spike protein from the unbiased simulations. The difference in the kinetics explains why we have been able to observe large-scale conformational changes in some of the SARS-CoV-1 spike protein simulations but not in any of the SARS-CoV-2 spike protein simulations. It is also important to note that the work analysis here does not provide much information on the thermodynamics. To be able to make statements about thermodynamics, we need to perform very accurate free-energy calculations.

Our SMD simulations show that it is relatively difficult for the CoV-2 spike protein to undergo a large-scale conformational transition between active and inactive states, when compared with the CoV-1 spike protein. Although these SMD simulations were run using the full trimers, they involved only a single protomer (protomer A) in the biasing schemes, whereas the other two protomers were not biased. These simulations were also repeated with all three protomers being biased (Fig. S14), which verified the large difference between the CoV-1 and CoV-2 kinetics. Our results indicate that the energy barriers associated with conformational changes that are required for activation and inactivation are larger in the CoV-2 spike protein as compared with CoV-1.

## Discussion

Using microsecond-level equilibrium and nonequilibrium MD simulations, we have demonstrated that the active CoV-1 and CoV-2 spike proteins exhibit differential dynamic behavior. The active CoV-2 spike protein remains relatively stable over 5 μs, whereas the active CoV-1 spike protein undergoes conformational changes and adopts, at least in one simulation, a pseudoinactive conformation that is distinct from the well-characterized inactive “RBD-down” conformation (38). Our observation of a pseudoinactive state of the CoV-1 spike protein essentially agrees with the results of an experimental smFRET study that describes alternative inactive states of the CoV-2 spike protein (48). While this pseudoinactive conformation is not observed in our CoV-2 spike protein simulations, it is certainly plausible that the CoV-2 spike protein samples alternate conformational states during the spike protein activation process that is dependent on the experimental/physiological conditions. In general, the key conclusion from the observation of this pseudoinactive state is that the published cryo-EM structures that are produced under nonphysiological conditions do not necessarily represent all relevant conformational states of the spike protein.

While our unbiased simulations provide some insight into the spike protein inactivation process, SMD simulations can access longer timescale conformational dynamics, which allows for a more detailed characterization of both activation and inactivation. In addition, nonequilibrium work measurements provide a semiquantitative method of comparing the energetics of the two proteins. An investigation of the energetics of the activation–inactivation process using SMD simulations revealed that relative to CoV-1, it is difficult for the CoV-2 spike protein to undergo a major conformational transition from the active state to the inactive state or vice versa. Nonequilibrium work measurements indicate that large-scale conformational transitions occur relatively slowly in the CoV-2 spike protein, which complements our observations on the relative conformational stability of the active CoV-2 spike protein from the equilibrium simulations, explaining the spontaneous conformational transition observed in the initially active CoV-1 equilibrium trajectory. The results from our equilibrium and nonequilibrium simulations are thus very consistent and provide extensive insights into the long-term dynamics of the CoV-1 and CoV-2 spike proteins. A recent computational study has shown that the RBD of the CoV-2 spike protein has greater mechanical stability than the RBD of the CoV-1 spike protein (58), which agrees with our observations on the conformational stability of the active CoV-2 spike protein.

Several cryo-EM studies have reported differing results on the propensity of the CoV-1 and CoV-2 spike proteins to adopt certain conformations (*e.g.*, one RBD “up” or three RBDs “down”). For instance, Kirchdoerfer *et al.* (41) stated that the single RBD “up” conformation is highly favored by the CoV-1 spike protein, with 58% of particles belonging to this population. They did not observe the three RBDs with “down” conformation (41). On the other hand, Yuan *et al*. (38) and Gui *et al*. (59) reported that particles in the three RBDs with “down” conformation make up approximately 56% and 27% of the population, respectively. Similarly, for the CoV-2 spike protein, Walls *et al*. (9) observed an approximately even split between the one RBD with “up” and three RBDs with “down” conformations, whereas Wrapp *et al.* (27) only observed the one RBD with “up” conformation. In our study, we do not make any claims about the predominance or relative stability of these conformations for the CoV-1 or CoV-2 spike protein. Instead, we focus exclusively on the differential dynamic behavior of the CoV-1-active and CoV-2-active spike proteins. Our study provides new insights into the kinetics, and not the thermodynamics, of the CoV-1 and CoV-2 spike protein activation process.

Using SPR and protein pull-down assays, Shang *et al.* (60) have shown that the CoV-2 spike RBD has significantly higher ACE2-binding affinity than the CoV-1 spike RBD. However, their results also indicate that the ACE2-binding affinity of the entire CoV-2 spike protein is similar to or lower than that of the CoV-1 spike protein (60). To explain this “paradox,” the authors hypothesize that although the CoV-2 has a higher-affinity RBD than CoV-1, the CoV-1 favors the up state of the RBD more than the CoV-2, and thus has a higher accessibility to ACE2. Since we do not make any claims regarding the thermodynamics (*i.e.*, up *versus* down stability), we can neither rule out nor provide evidence for this hypothesis based on our simulations. However, given the fact that the spike–ACE2 binding is only the first step in a cascade of events that result in S1–S2 cleavage and membrane fusion, it is important for the spike–ACE2 association to last long enough so the rest of the process is triggered. Therefore, kinetics is perhaps as important if not more important than thermodynamics here. Our hypothesis based on the slower kinetics is that once the SARS-CoV-2 spike protein is activated, not only is it ready to bind to ACE2 but also it favors staying bound for a long enough time such that a cascade of events necessary for membrane fusion can occur.

Unlike X-ray crystallography and cryo-EM, MD simulations facilitate the elucidation of detailed hypotheses on the dynamic behavior of proteins and other biomolecules (46, 47). However, each computational or experimental technique has its own assumptions and limitations. Here, for instance, we chose to work with the nonglycosylated spike proteins of CoV-1 and CoV-2 to avoid complications when making comparisons. A recent study has shown that glycosylation of the spike proteins might play an important role in the conformational dynamics of the RBD (61, 62). At this stage, we have not simulated the glycosylated spike proteins because of the difficulty of modeling the correct glycan chains. It would be quite difficult to determine whether conformational changes occur as a result of the intrinsic protein dynamics or the differential glycosylation patterns of the CoV-1 and CoV-2 spike proteins imposed by our modeling. However, we use the nonglycosylated form of the spike protein for both CoV-1 and CoV-2, which makes the comparison justifiable.

Investigation of the “effective binding” process involving both receptor interaction and spike protein activation will provide deeper insights into the enhanced infectivity of SARS-CoV-2. Several studies have investigated RBD–ACE2 binding for both SARS CoV-1 (63, 64, 65, 66, 67, 68, 69) and SARS-CoV-2 (9, 17, 27, 36, 37), while ignoring the conformational dynamics of spike protein activation and inactivation. We propose that the “effective binding” process is different in the CoV-1 and CoV-2 spike proteins, not only because of the variability of the RBD but also because of the contribution of other regions, particularly the NTD, as seen in the CoV-1 pseudoinactive state, where the NTD interacts with RBD and therefore could block the ACE2 binding to RBD. This is in qualitative agreement with the results of recent experimental and clinical studies, which highlight the importance of the spike protein NTD in the SARS-CoV-2 infection process (70, 71, 72, 73, 74, 75).

Several circulating SARS-CoV-2 variants with mutations or deletions in the NTD show greatly reduced recognition by NTD-specific neutralizing monoclonal antibodies (70, 71, 72, 73, 74, 75). This strongly suggests that the NTD is under selective pressure from the host humoral immune response (70, 71, 72, 73, 74, 75). Based on the observation of a previously unknown pseudoinactive conformational state for the spike protein, we hypothesize that the RBD–NTD interaction could play a crucial role in the inactivation of the spike protein and that mutations in the spike NTD could potentially have an effect on the transmissibility of the coronavirus. A recent study has shown that the breakage of several RBD–S2 electrostatic interactions is required for S1–S2 dissociation (76). As observed in our simulations, one of these interactions involves a conserved residue pair that forms a salt bridge between the RBD of the active protomer and the S2 region in the CoV-2 spike protein (R319 and D745) but not in the CoV-1 spike protein (R306 and D727). This interaction thus contributes to the relative stability of the active CoV-2 spike protein and the differential dynamic behavior observed in our simulations. While the vast majority of studies focus on the RBD for obvious reasons, the functional relevance of other regions like the NTD and S2 needs to be investigated in greater detail using both experimental and computational methods. More generally, our simulations suggest that the differential conformational dynamics associated with inactivation and activation of the coronavirus spike protein might contribute to the increased transmissibility of SARS-CoV-2 as compared with SARS-CoV-1 and some variants of SARS-CoV-2 as compared with some other variants. It is important to note that like any other experimental or computational technique, all-atom MD has its own inherent limitations, requiring the use of other techniques to validate its results. Most importantly, the timescales of our simulations, although consistent with the state of the art, are still shorter than those associated with the full activation process of the spike protein. Fortunately, our simulations have been long enough to reveal the differential behavior of the CoV-1 and CoV-2 spike proteins in their prefusion state as detailed previously. More importantly, these simulations have been extensive enough to point us to new hypotheses that are experimentally testable.

Several experiments could be performed in order to test the hypotheses presented in our computational study. For instance, the importance of residues D23 and D24 from the CoV-1 spike NTD could be investigated *via* site-directed mutagenesis. This might provide some additional insights on the conformational dynamics of the CoV-1 spike protein. Similarly, the conserved residue pairs that exhibit differential behavior in terms of salt-bridge interactions could be mutated in both spike proteins. In addition, smFRET experiments could be used to investigate a potential RBD–NTD interaction by measuring the distance between fluorophores attached to each domain. Disulfide crosslinking experiments could also be used to investigate residues in the NTD and RBD that potentially interact with each other.

As discussed previously, our study primarily sheds light on the conformational dynamics of the SARS-CoV-1 and SARS-CoV-2 spike proteins. While differences in the dynamic behavior of these spike proteins almost certainly contribute to differences in transmissibility and infectivity, factors such as spike protein glycosylation and the behavior of other viral proteins also need to be considered in order to provide a more complete hypothesis. Additional experimental and computational studies are thus needed to fully investigate the differential infectivity and transmissibility of SARS-CoV-1 and SARS-CoV-2.

## Experimental procedures

Our simulations were based on cryo-EM structures of the SARS-CoV-2 spike protein in the active (Protein Data Bank [PDB] entry: 6VYB) (9) and inactive (PDB entry: 6VXX) (9) states and the SARS-CoV-1 spike protein in the active (PDB entry: 5X5B) (38) and inactive (PDB entry: 5X58) (38) states. The protein was solvated in a box of transferable intermolecular potential 3 point water with 0.15 M NaCl and was simulated using CHARMM36m all-atom additive force field (77). For details of our simulation and analysis methods, see Supporting information.

## Data availability

Simulation and analysis scripts are available on GitHub-https://github.com/bslgroup/Spike_Protein.

## Supporting information

This article contains supporting information.

## Conflict of interest

The authors declare that they have no conflicts of interest with the contents of this article.
