# Supplementary material for: Prefusion spike protein conformational changes are slower in SARS-CoV-2 than in SARS-CoV-1
Source: J Biol Chem. 2022 Mar 10;298(4):101814. doi: 10.1016/j.jbc.2022.101814 (PMC8907130; doi:10.1016/j.jbc.2022.101814)
Supplement: Supplemental Information [file mmc2.pdf]

Supporting Information: Prefusion Spike Protein Conformational Changes are Slower in SARS-CoV-2 than in SARS-CoV-1

Vivek Govind Kumar, Dylan S Ogden, Ugochi H Isu, Adithya Polasa, James Losey, and Mahmoud Moradi*

Department of Chemistry and Biochemistry, University of Arkansas, Fayetteville, Arkansas 72701, U.S.A.

E-mail: moradi@uark.edu

Simulation and Analysis Details

MD Simulation Details

We have used all-atom equilibrium and nonequilibrium MD simulations to characterize the conformational dynamics of the spike protein from SARS-CoV-2 and SARS-CoV-1. Our simulations were based on cryo-EM structures of the SARS-CoV-2 spike protein in the active (PDB entry:6VYB)[1] and inactive (PDB entry:6VXX)[1] states and the SARS-CoV-1 spike protein in the active (PDB entry:5X5B)[2] and inactive (PDB entry:5X58)[2] states. Missing residues for all 4 models were generated using Modeller.[3] 10,000 Monte Carlo iterations were used to generate the initial models for the equilibrium simulations.[3] CHARMM-GUI[4,5] was then used to build the simulation systems. Engineered residues were mutated back to the wildtype and disulfide bonds were added to each model based on the information provided in the respective PDB files.[1,2] The protein was solvated in a box of TIP3P waters, and 0.15 M NaCl (in addition to the counterions used to neutralize the protein) using CHARMM-GUI.[4,5]

All simulations were performed using the NAMD 2.13[6] simulation package with the CHARMM36m all-atom additive force field.[7] The input files for energy minimization and production were generated using CHARMM-GUI.[4,5] Initially, we energy-minimized each system for 10,000 steps using the conjugate gradient algorithm.[8] Then, we relaxed the systems using restrained MD simulations in a stepwise manner using the standard CHARMM-GUI protocol[4,5] ("relaxation step"). In the next step, backbone and sidechain restraints were used for 10 ns with a force constant of 1 kcal/mol.$Å^2$ and 0.5 kcal/mol.$Å^2$, respectively ("restraining step"). The systems were then equilibrated with no bias for another 10 ns ("equilibration step"). The initial relaxation was performed in an NVT ensemble while the rest of the simulations were performed in an NPT ensemble. Simulations were carried out using a 2-fs time step at 310 K using a Langevin integrator with a damping coefficient of $\gamma = 0.5$ ps$^-1$. The pressure was maintained at 1 atm using the Nose-Hoover Langevin piston method.[8,9] The smoothed cutoff distance for non-bonded interactions was set at 10 to

12 Å and long-range electrostatic interactions were computed with the particle mesh Ewald (PME) method.[10]

These initial simulations were executed on TACC Longhorn. The production run for each model was then extended to 5 μs on Anton2,[11] with a timestep of 2.5 fs. Conformations were collected every 240 picoseconds. Initial processing of the Anton2 simulation trajectories was carried out on Kollman.[11] Two additional 5 μs simulations were performed for both the CoV-2 and CoV-1 active models on Anton2 (referred to as Set 2 and Set 3 in the manuscript). As stated previously, cryo-EM structures (PDB entries:6VYB,5X5B)[1,2] were used as starting conformations for each model. In order to generate initial conformations for Set 2, the original production run ("equilibration step" described previously) for each model was extended by 0.5 ns on TACC Longhorn. The production runs were then extended again by 0.5 ns to generate the initial conformations for Set 3. 40 μs of simulation data was generated in aggregate – 15 μs each for the active Cov-1/Cov-2 spike proteins and 5 μs each for the inactive spike proteins.

RBM-S2 Distance and Angle.

To quantify the RBM-S2 distance, we defined centers of mass based on residues that form a beta-sheet in the RBM region of each RBD (CoV-1: RBM residues 439 to 441, 479 to 481; CoV-2: RBM residues 452 to 454, 492 to 494) and residues that encompass the S2 trimer (CoV-1: S2 residues 672 to 1104; CoV-2: S2 residues 690 to 1147). We then measured the vector distance between the two centers of mass and used the vector magnitude to quantify the overall distance.

For the RBM-S2 angle, we chose residues at the top and bottom of the straightest region of the S2 Trimer (alpha-helical regions in CoV-1: residues 970 and 1016; CoV-2: residues 914 and 987). Similarly, we also chose residues from the beta-sheet region of the RBM and one at the bottom of the RBD (CoV-1: residues 348 and 478; CoV-2: residues 391 and 493). We then defined a vector direction using the vector subtraction of the two chosen residues

in the S2 region and the residues of the RBD region, which were defined as v_1 and v_2. The vector angle between the RBD and S2 was then calculated with the following equation: $\arccos(\frac{v_1 \cdot v_2}{|v_1||v_2|})$. The computed angle was subtracted from 180°. An angle above ≈60° would indicate an RBD in the inactive conformation with respect to S2, and 0-40° would indicate an RBD in the active conformation.

NTD-RBD Distance

To characterize conformational changes in the active and inactive states of both CoV-1 and CoV-2 spike proteins, we calculated the minimum distance between every residue of the receptor-binding domain (RBD) and the N-terminal domain (NTD). We measured the distance between each residue pair in these regions (maximum distance cut off was 20 Å) as a function of time. The domains were defined as follows: CoV-2 RBD (residues 330 to 515); CoV-2 NTD (residues 60 to 270); CoV-1 RBD (residues 330 to 550); CoV-1 NTD (residues 35 to 255).

RBM Hydration Analysis.

The amount of solvent around the receptor-binding motif (RBM) was quantified using a VMD[12] script. We calculated the number of water molecules within 5 Å of the RBM for every frame of the last 500 ns of each trajectory and also plotted probability density maps for each water count.

Principal Component Analysis (PCA).

PCA[13] performed with ProDy[14] was used to quantify the persistent conformational changes and relative motions of the active and inactive states. Only the position of the C-α atoms of the spike protein was considered when building the covariance matrix of atomic positions, in order to focus on the large conformational changes and ignore side chain fluctuations.

Each trajectory was aligned with the positions from the cryo-EM structure before analysis to remove translational motion of the protein from the variance calculations.

The CoV-1/CoV-2 active state (Set 1) and CoV-1/CoV-2 inactive state trajectories were stripped down to trajectories of the individual protomers from each simulation. The individual protomers were then analyzed together to compare and quantify the relative motions of the active and inactive states. Through eigenvalue decomposition, the top twenty principal components (PCs) were calculated for each protomer. The top two PCs for each protomer have been plotted to identify the major motions of the protein.

Dynamic Network Analysis (DNA).

DNA[15] of the correlated motions of the protein provided further quantitative information on the concerted motions of the C-α atoms of the protein. MD-TASK,[16] a software suite of MD analysis tools, was used to calculate the correlation coefficient for the motion of each C-α atom relative to the other C-α atoms. A correlation matrix M was generated for each of the three protomers in all the simulated trajectories. Additionally, a correlation matrix for the entire trimer was calculated for each simulation to explore correlations between structures of different protomers. A step size of four frames was used during the correlation calculations to reduce the processing times, given the large number of residues.

To quantify the differences in correlation between a protomer and some reference, a difference matrix, Δ was calculated,

$$\Delta = |M_i - M_{Ref.}|, \tag{1}$$

where M_i is the correlation matrix of interest, and M_{Ref} is the correlation matrix of a reference conformation. In this work, the difference between a protomer in an active conformation and an inactive conformation was of interest. For this reason, the protomers in the active simulations were compared with Protomer C in the inactive simulation, which displayed

relatively little motion.

Interaction Analysis.

To identify interactions that contribute to the stability of the Cov-2 spike protein or play key roles in the CoV-1 active conformational transition, we performed salt-bridge and hydrogen-bond analysis for all SARS-CoV-2 and SARS-CoV-1 systems. Salt bridges were identified using the VMD Timeline plugin[12] at a cutoff distance of 4.0 Å. The salt-bridge cutoff distance is defined as the distance between the oxygen atom of the participating acidic residue and the nitrogen atom of the basic residue. The VMD HBond plugin[12] was used for hydrogen bond analysis. The donor-acceptor distance and angle cutoffs used were 3.5 Å and 30 degrees respectively. We report salt-bridge and hydrogen-bond interactions that illustrate the differential behavior of the SARS-CoV-2 and CoV-1 spike proteins.

Steered Molecular Dynamics (SMD) Analysis.

To induce activation/inactivation of a protomer initially in the inactive/active conformation, we defined collective variables based on the the Cα RMSD of each protomer in the CoV-1 and CoV-2 systems. Reference coordinates were taken from the corresponding active/inactive structure for both CoV-1 and CoV-2 protomers. The atoms chosen were based on the total number modeled residues in the CoV-2 structures. Structural analysis of CoV-1 and CoV-2 was employed to ensure that equivalent Cα atoms were steered in all simulation sets. 1037 atoms were steered for any given protomer and the following residue range was used: 27 to 239, 244 to 315, 322 to 662, 673 to 809, and 831 to 1104. These atoms span the entire protomer, starting from the NTD and ending approximately at the C-terminus of the S2 region. A force constant of 250 $kcal/mol/Å^2$ was used for SMD simulations involving a single protomer and a force constant of 750 $kcal/mol/Å^2$ was used for SMD simulations involving all three protomers. The systems used for each simulation were taken from the outcome of the "equilibration step" as explained above. Utilizing the multi-copy capabilities

of NAMD, we performed 10 sets of 100 ns RMSD steering for each system - 8μs of simulation time in aggregate.

For all SMD time series analyses, each data point was averaged for the 10 sets and standard deviation was calculated. Each analysis was plotted with 100 points and error bars were derived from the standard deviation. The RBM-S2 distance and angle calculations were performed as described previously. Using the Jarzynski relation[17] we calculate the Jarzynski average at time t during the activation or inactivation process as $-k_B T \ln \sum_{i=1}^{N} \exp\left(\frac{-W_i(t)}{k_B T}\right)/N$, where k_B and T are the Boltzmann constant and the temperature, respectively and $W_i(t)$ is the work accumulated from the beginning of the SMD simulation i up to time t. The above average would converge to the free energy for large number of trajectories ($N \rightarrow \infty$). For $N = 10$, the above average simply provides a semi-quantitative measure for relative energetic comparisons.[18–21]

Supporting Discussion: Principal Component Analysis and Dynamic Network Analysis

We performed principal component analysis (PCA) to validate our claim that the active form of the CoV-2 spike protein is more stable than the active CoV-1 spike protein and to provide insight into the mechanistic aspects of the spike protein activation-inactivation process. When the individual protomer trajectories (see Methods section) from the CoV-1/CoV-2 active (Set 1) and inactive simulations are projected onto the space of their first two principal components (PC1 and PC2), it clearly demonstrates that the CoV-1 active protomer A samples a much larger region in the PC1 space than CoV-2 active protomer A (Figures S3A, S3C). This is further evidence of the relative stability of the active CoV-2 spike protein in comparison to the active CoV-1 spike protein.

A visual representation of PC1 for all protomers from the CoV-1 spike protein simulations shows that the RBD undergoes the most pronounced motions directed inward towards the NTD (Figure S3B). On the other hand, a visual representation of PC1 for the CoV-2 spike protein shows that the RBD and NTD tend to move away from each other slightly and that the fluctuations are significantly smaller than in the CoV-1 spike protein (Figure S3D). The most pronounced collective motion in each system (PC1) describes the distinct motions associated with the RBD, that play key roles in the inactivation of the active CoV-1 spike protein and maintenance of the active conformation of the CoV-2 spike protein (Figure 1). This highlights the differential dynamic behavior of the active CoV-1 spike protein.

PC2 describes the relative motions of the NTD and RBD, showing that the NTD motion is more pronounced in CoV-1 (Figure S4). The motions associated with PC2 are roughly the opposite of those associated with PC1 in terms of direction. PC2 also shows that the CoV-1 spike protein has more regions outside the NTD and RBD that show high variance (Figure S4). Similar trends are observed in Sets 2 and 3 of the active state simulations (Figure S5). While different protomers are involved, the active CoV-1 spike protein still undergoes

more pronounced motions in both PC1 and PC2 compared to the active CoV-2 spike protein (Figure S5). These observations are in agreement with our claim that the active CoV-2 spike protein is relatively stable and that the active CoV-1 spike protein transitions spontaneously to a pseudo-inactive conformation.

The inferences drawn from PCA are also supported by dynamic network analysis (DNA). Differential behavior of the active CoV-1 and CoV-2 spike proteins manifests in the correlation of motions between the various domains in individual protomers. In Figure S6A, correlation heat maps of active CoV-1 protomer A (Set 1) and inactive CoV-1 protomer C are presented, along with the difference between the active state and the reference structure (inactive protomer C). The heat map for active Cov-1 protomer A shows regions of high correlation and anticorrelation between several domains of the protomer. The NTD correlates strongly with itself while anticorrelating with the RBD and parts of the S2 region. The reference protomer, inactive CoV-1 protomer C, shows a general reduction in correlation across all regions (Figure S6A). The NTD does correlate with itself, but not as strongly as in the active CoV-1 protomer A.

Similarly, the NTD-RBD anticorrelations were reduced. The Δ matrix of differences between active CoV-1 protomer A and inactive protomer C identified the regions where the correlations were most different. Correlations between S1-C and the NTD/RBD changed significantly, as did correlations between the RBD and S2 region (Figure S6A).

The correlations and anti-correlations observed for active CoV-2 protomer A (Set 1) were not as strong as those observed for active CoV-1 protomer A (Figure S6B). Similar to CoV-1, anti-correlation occurs between the NTD and RBD but is not as pronounced. Very low correlation was observed between the NTD and S1-C/S2 regions, also differentiating CoV-2 from CoV-1. The active CoV-2 protomer A is closer to the stable inactive CoV-2 protomer C, as shown in the Δ matrix (Figure S6B). DNA correlation heat maps for all protomers in Set 1 of the CoV-1/CoV-2 active state simulations are shown in Figures S7 and S8 respectively. Similar trends were observed in Set 2 and Set 3 of the CoV-1 and CoV-2

active state simulations (Figure S9-S10). These observations thus provide further evidence of the relative stability of the active CoV-2 spike protein.

The concerted movements of each protomer relative to the rest of the trimer also highlight the differences between the active CoV-1 and CoV-2 spike proteins. Heat maps showing correlations between NTD regions of different protomers are presented in Figure S11A. Stronger correlations and anticorrelations occurred in Sets 2 and 3 of the active CoV-1 simulations (Figure S11A). Set 2 showed moderately strong anticorrelations between NTDs A-C and NTDs B-C. Stronger anti-correlations between NTDs A-B and NTDs B-C occurred in Set 3, with moderate correlations between NTDs A-C. The active CoV-2 simulations showed similar correlations across all three simulation sets, with slightly increased values in Set 3 (Figure S11A). These observations are consistent with a more stable conformation for the active CoV-2 spike protein.

Figure S11B shows a similar trend with correlations between the NTD and RBD regions of different protomers. Sets 2 and 3 of the active CoV-1 spike protein trajectories showed stronger correlations between the NTD and RBD regions than the corresponding CoV-2 trajectories (Figure S11B). In particular, RBD C of Sets 2 and 3 had strong correlations or anticorrelations with the NTDs of all protomers (Figure S11B). The CoV-2 simulations displayed lower correlations for all the NTD-RBD combinations, with similar results for both active state and inactive state trajectories (Figure S11B). This recapitulates our other observations of greater conformational stability of the active CoV-2 spike protein relative to the active CoV-1 spike protein (Figures 1, S3, S6).

Supporting Figures

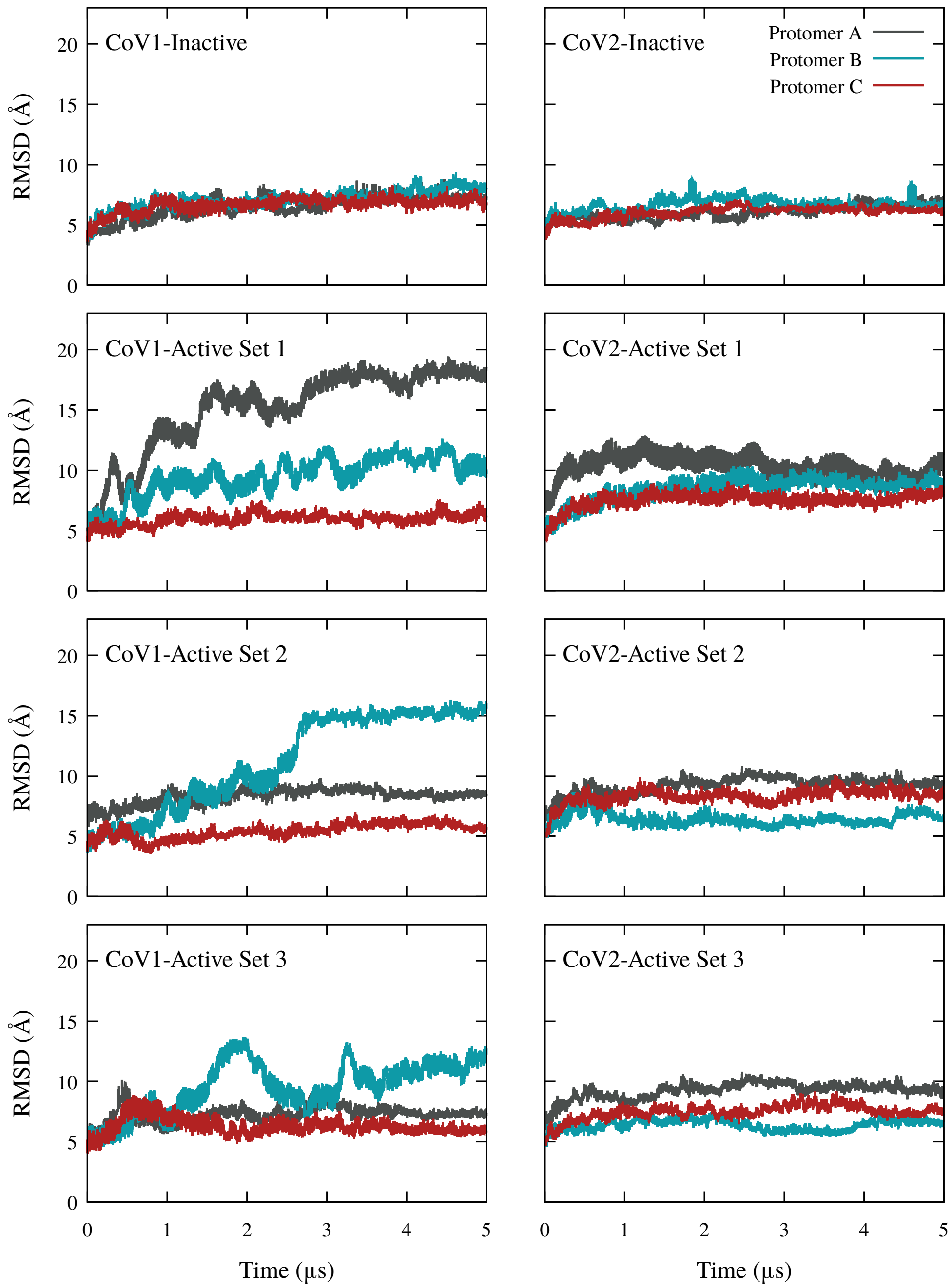

Figure S1: C-α RMSD for individual protomers. The C-α RMSD calculated for each protomer relative to the initial cryo-EM structure over the 5 μs simulation is plotted for the inactive spike simulations and three sets of active spike simulations. Protomer A is colored dark grey, protomer B is colored light blue, and protomer C is colored dark red. The active CoV-2 spike protein is more stable overall than the active CoV-1 spike protein.

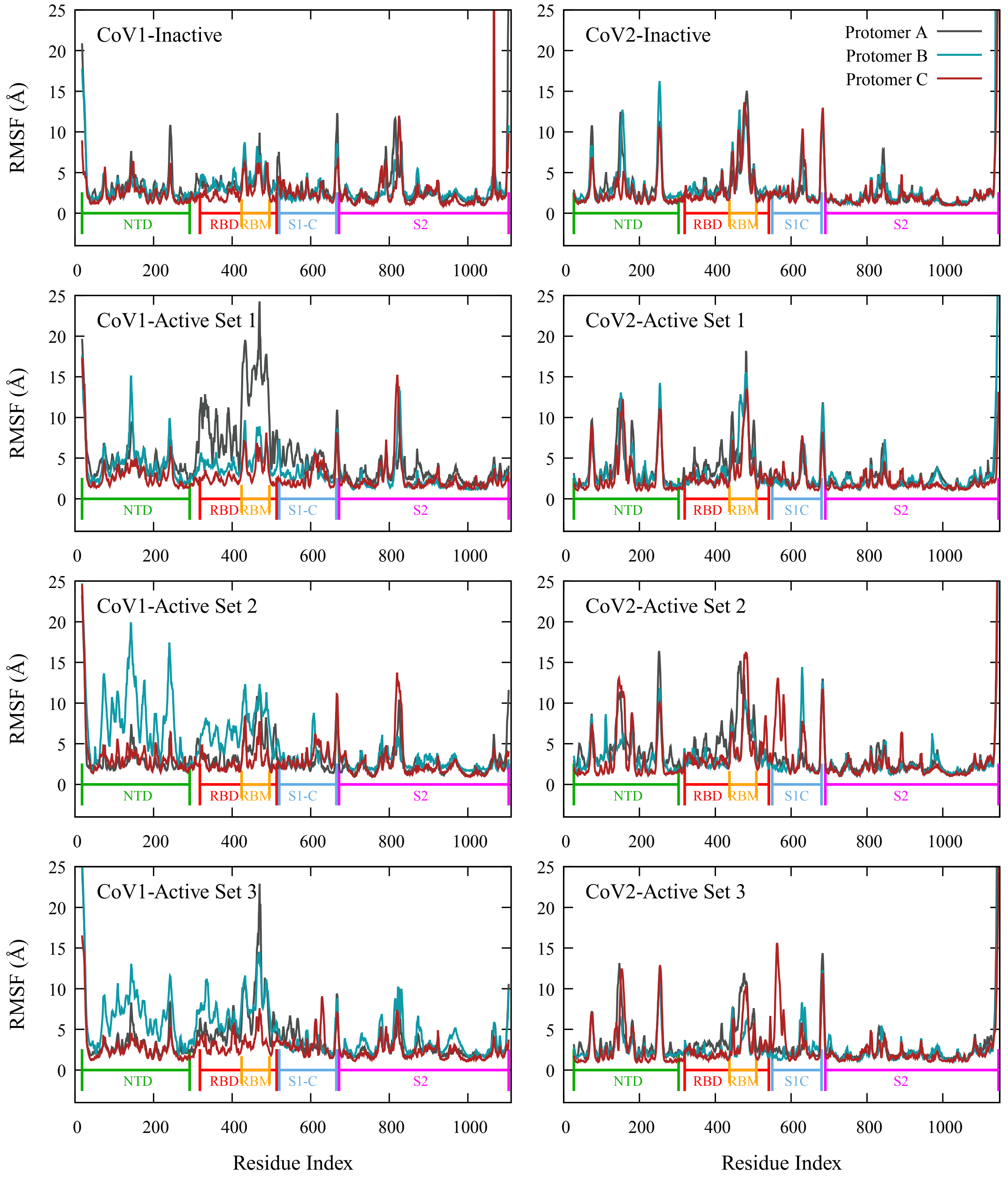

Figure S2: **C-α RMSF for individual protomers.** The C-α RMSF for each protomer relative to the initial cryo-EM structure position was calculated for the inactive spike simulations and three sets of active spike simulations. Protomer A is colored dark grey, protomer B is colored light blue, and protomer C is colored dark red. The NTD and RBD of the active CoV-1 spike are more flexible than the corresponding regions of the active CoV-2 spike.

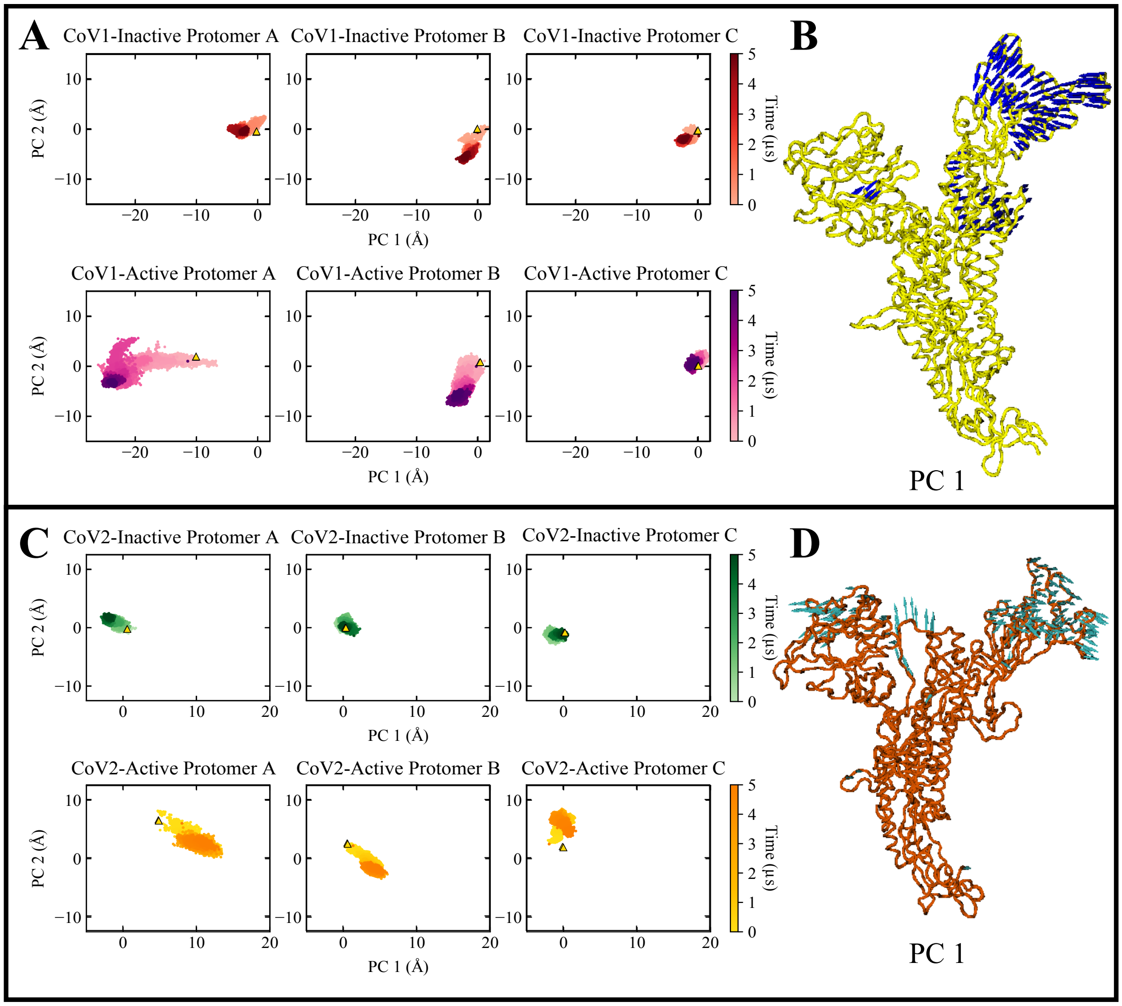

Figure S3: **Principal component analysis demonstrates that the active CoV-2 spike protein is more stable than the active Cov-1 spike protein.** **(A)** Scatter plot of PC1 and PC2 for each protomer in the active and inactive CoV-1 simulations. Protomers from inactive state simulations are colored red while protomers from active state simulations are colored magenta. Lighter/darker colors represent earlier/later stages in the simulation. **(B)** Visual representation of PC1 with the blue arrows at each C-α atom indicating direction and magnitude of variance. The RBD of the CoV-1 spike protein shows pronounced motions in the direction of the NTD. **(C)** Scatter plot of PC1 and PC2 for each protomer in the inactive and active CoV-2 simulations. Protomers from inactive state simulations are colored green while protomers from active state simulations are colored yellow. The active CoV-2 spike protein is relatively stable and samples significantly fewer conformations in the PC1 space in comparison to the active Cov-1 spike protein. **(D)** Visual representation of PC1 with the cyan arrows at each C-α atom indicating direction and magnitude of variance. The NTD and RBD of the CoV-2 spike protein show slight movement away from each other.

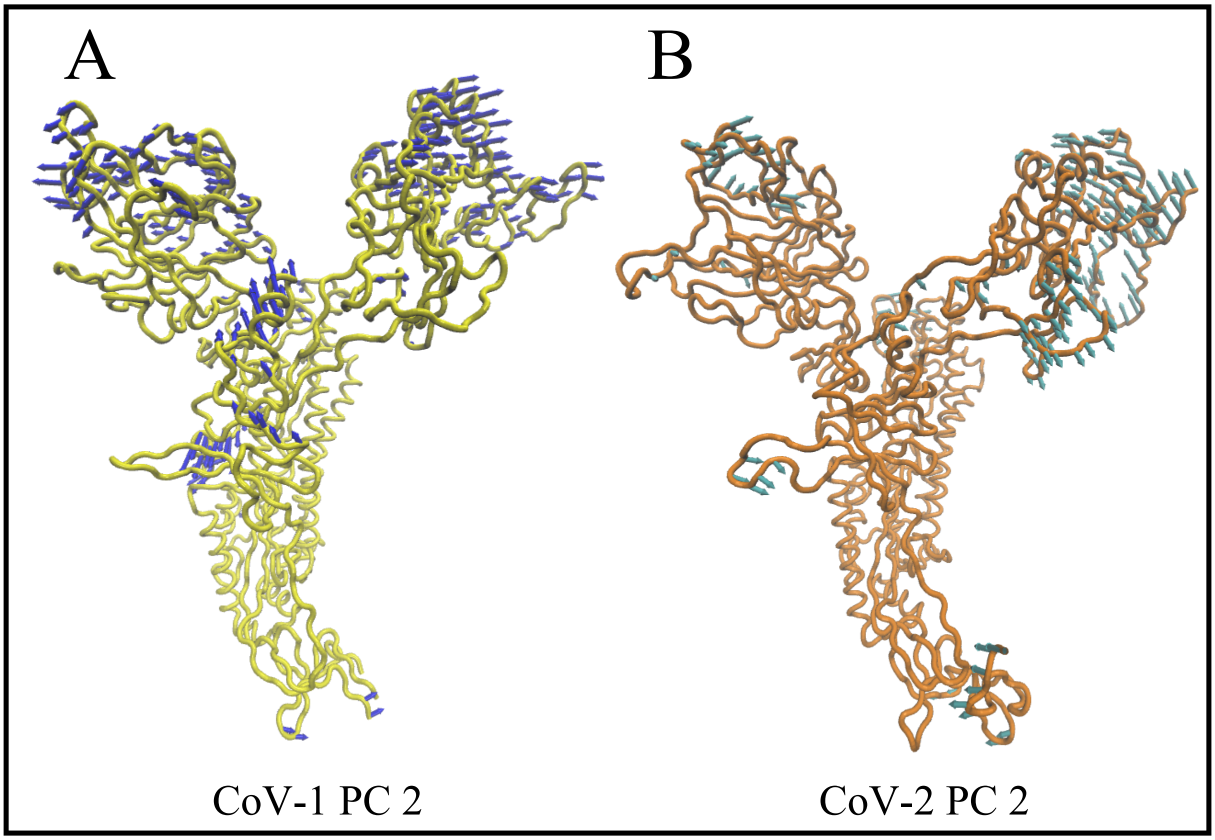

Figure S4: **Visual representation of PC2 for all protomers in the inactive and active (Set 1) spike simulations for CoV-1 and CoV-2. (A)** Visual representation of PC2 for all CoV-1 protomers with the blue arrows at each C-α atom indicating direction and magnitude of variance. **(B)** Visual representation of PC2 for all CoV-2 protomers with the blue arrows at each C-α atom indicating direction and magnitude of variance. The NTD motions contribute more to the conformations sampled in the PC2 space than the PC1 space. These NTD motions are more pronounced in the CoV-1 spike, which also has more regions outside the NTD/RBD that show high variance.

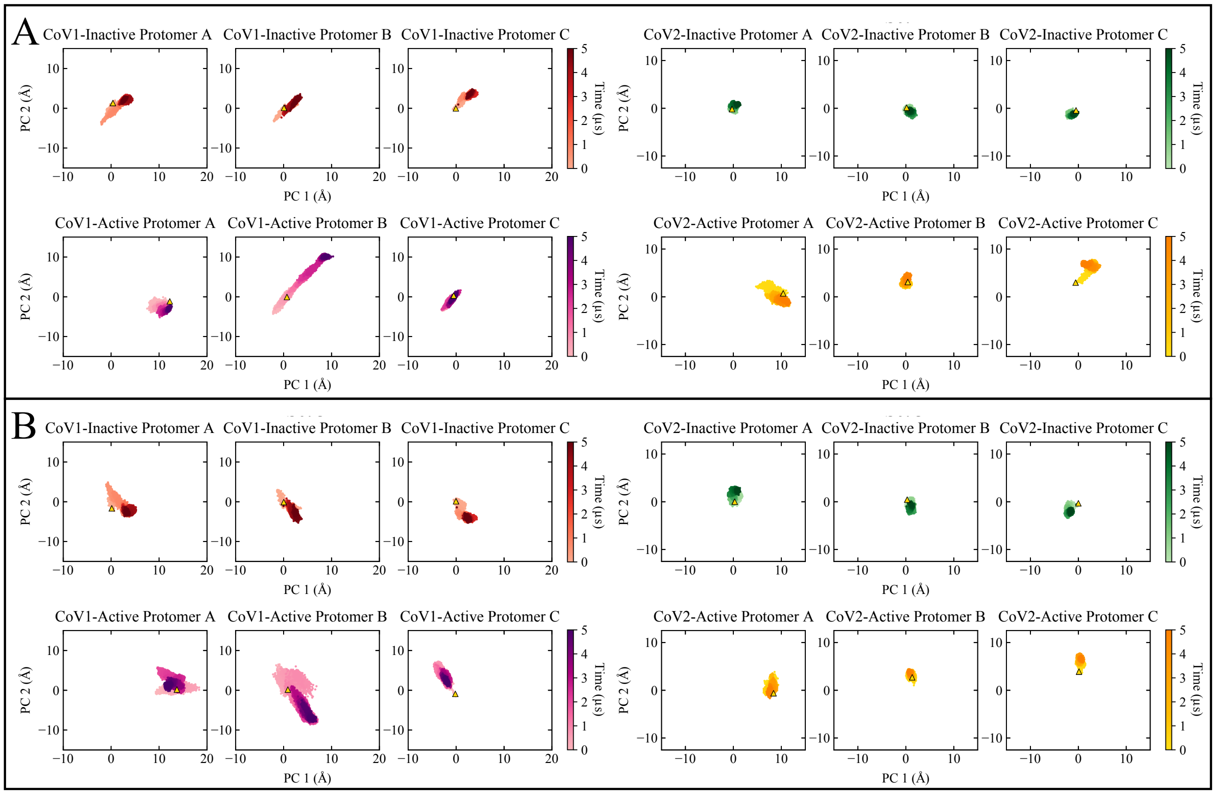

Figure S5: PCA of all protomers in the inactive and active (Sets 2 and 3) simulations for CoV-1 and CoV-2. (A) Scatter plot of PC1 and PC2 for Set 2 of CoV-1 and CoV-2 active and inactive spike simulations.**(B)** Scatter plot of PC1 and PC2 for Set 3 of CoV-1 and CoV-2 active and inactive spike simulations. The coloring is the same as seen in Figure 3 with darker shades representing frames towards the end of the simulations. The active CoV-2 spike clearly samples fewer conformations in both PC1 and PC2 spaces than the active CoV-1 spike.

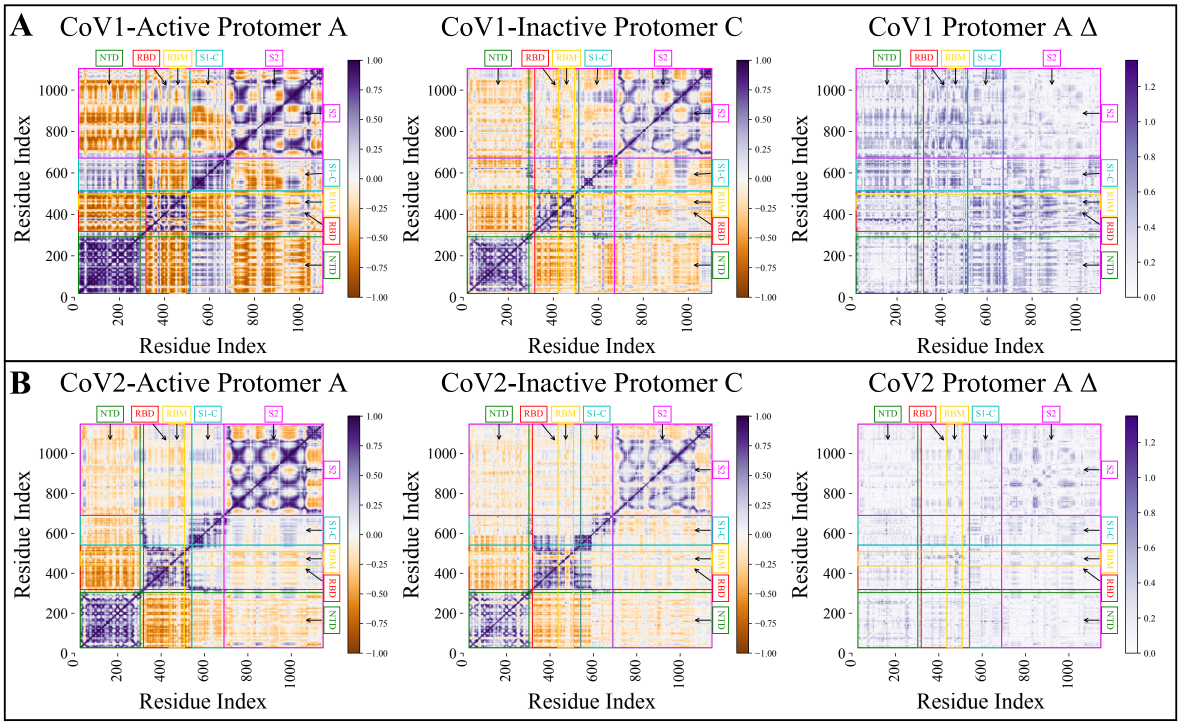

Figure S6: **Dynamic network analysis shows that intra-protomer correlations and anticorrelations are relatively strong in the active CoV-1 spike protein simulations.** **(A)** DNA heat maps showing the correlation of motions for the active CoV-1 protomer A, inactive protomer C (reference), and the difference matrix. **(B)** DNA heat maps showing the correlation of motions for the active CoV-2 protomer A, inactive protomer C (reference), and the difference matrix. Correlations are shown in purple and anti-correlations are shown in orange, with the darker colors indicating greater correlation/anti-correlation. Colored labels for the NTD (green), RBD (red), RBM (yellow), S1-C (cyan), and S2 (magenta) regions are positioned over the appropriate residues. The delta matrix identifies differences in protomer correlation between the active and reference inactive protomer. A theoretical maximum for Δ is 2, but the observed maximum was less than 1.3. Differences in correlation are shown as a purple gradient with darker purple indicating larger difference.

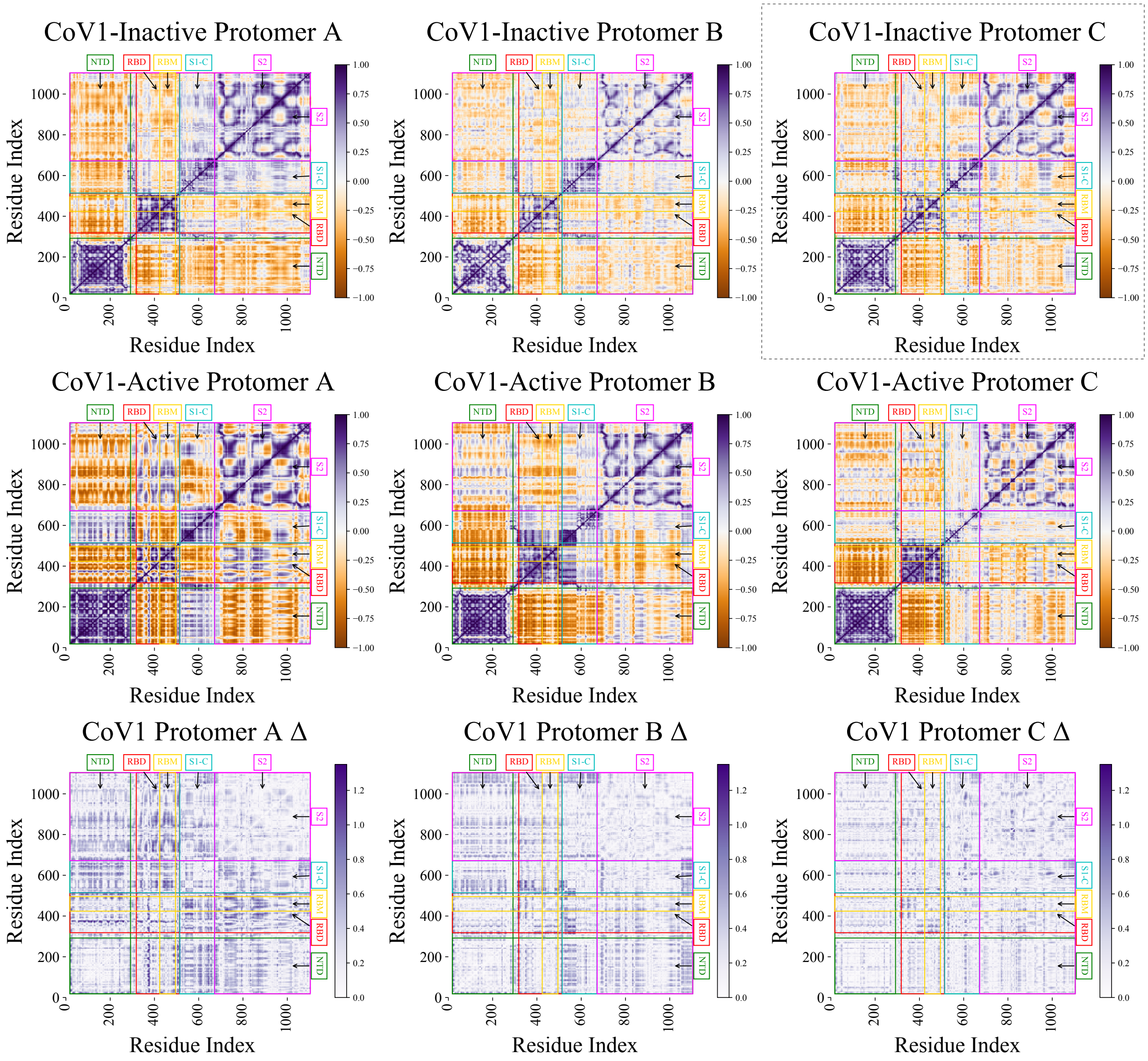

Figure S7: DNA correlation heat maps and Δ matrix for all protomers from the CoV-1 inactive and CoV-1 active (Set 1) spike simulations. DNA heat maps showing the correlation of motions for the CoV-1 inactive protomers (first row), the CoV-1 active protomers from Set 1 (second row) and the difference matrices. The inactive protomer C correlation matrix, indicated by the dotted box, is the reference used for calculating the Δ matrix. Panels corresponding to CoV1-Active Protomer A and CoV1-Inactive Protomer C are also shown in Fig. S6A to calculate their relative Δ matrix, which is different from Δ matrices shown here.

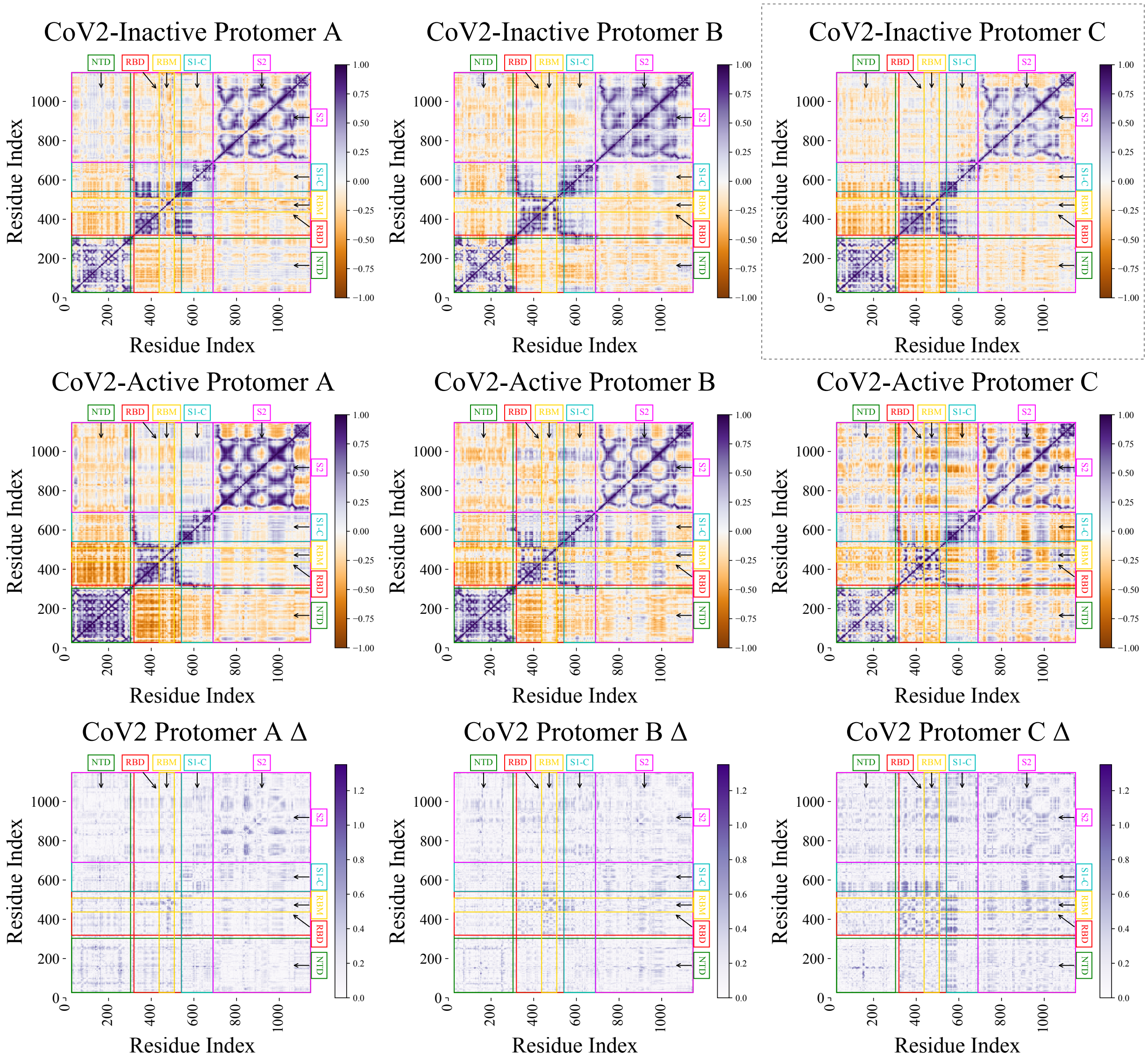

Figure S8: DNA correlation heat maps and Δ matrix for all protomers from the CoV-2 inactive and CoV-2 active (Set 1) spike simulations. DNA heat maps showing the correlation of motions for the CoV-2 inactive protomers (first row), the CoV-2 active protomers from Set 1 (second row) and the difference matrices. The inactive protomer C correlation matrix, indicated by the dotted box, is the reference used for calculating the Δ matrix. Panels corresponding to CoV2-Active Protomer A and CoV2-Inactive Protomer C are also shown in Fig. S6B to calculate their relative Δ matrix, which is different from the Δ matrices shown here.

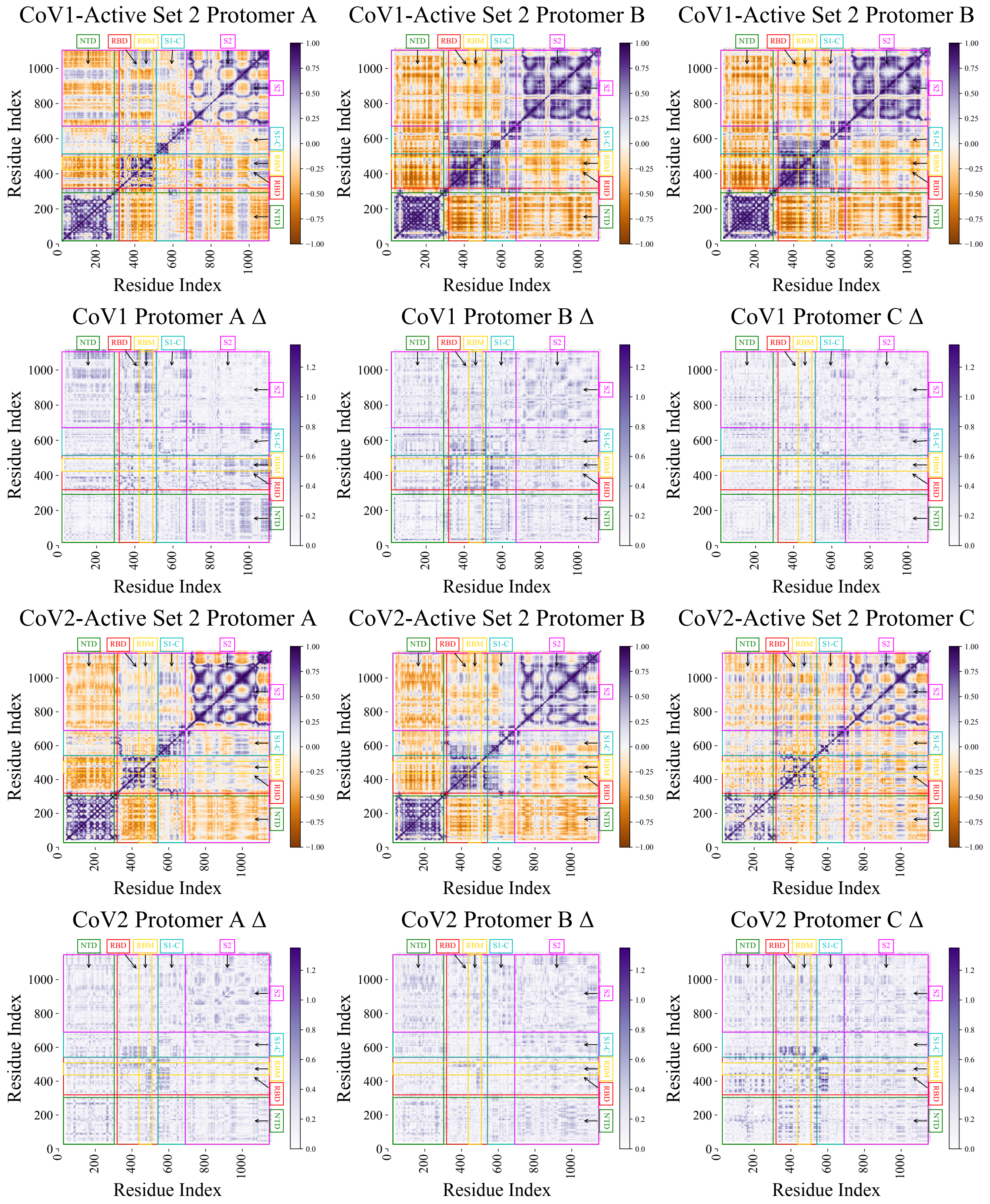

Figure S9: **DNA correlation heat maps and Δ matrix for all protomers from Set 2 of the CoV-1 and CoV-2 active spike simulations. (A)** DNA heat maps showing the correlation of motions for the CoV-1 active (Set 2) protomers (first row) and the difference matrices (second row). The reference matrix from Figure S7 was used for Δ matrix calculations. **(B)** DNA heat maps showing the correlation of motions for the CoV-2 active (Set 2) protomers (third row) and the difference matrices (fourth row). The reference matrix from Figure S8 was used for Δ matrix calculations.

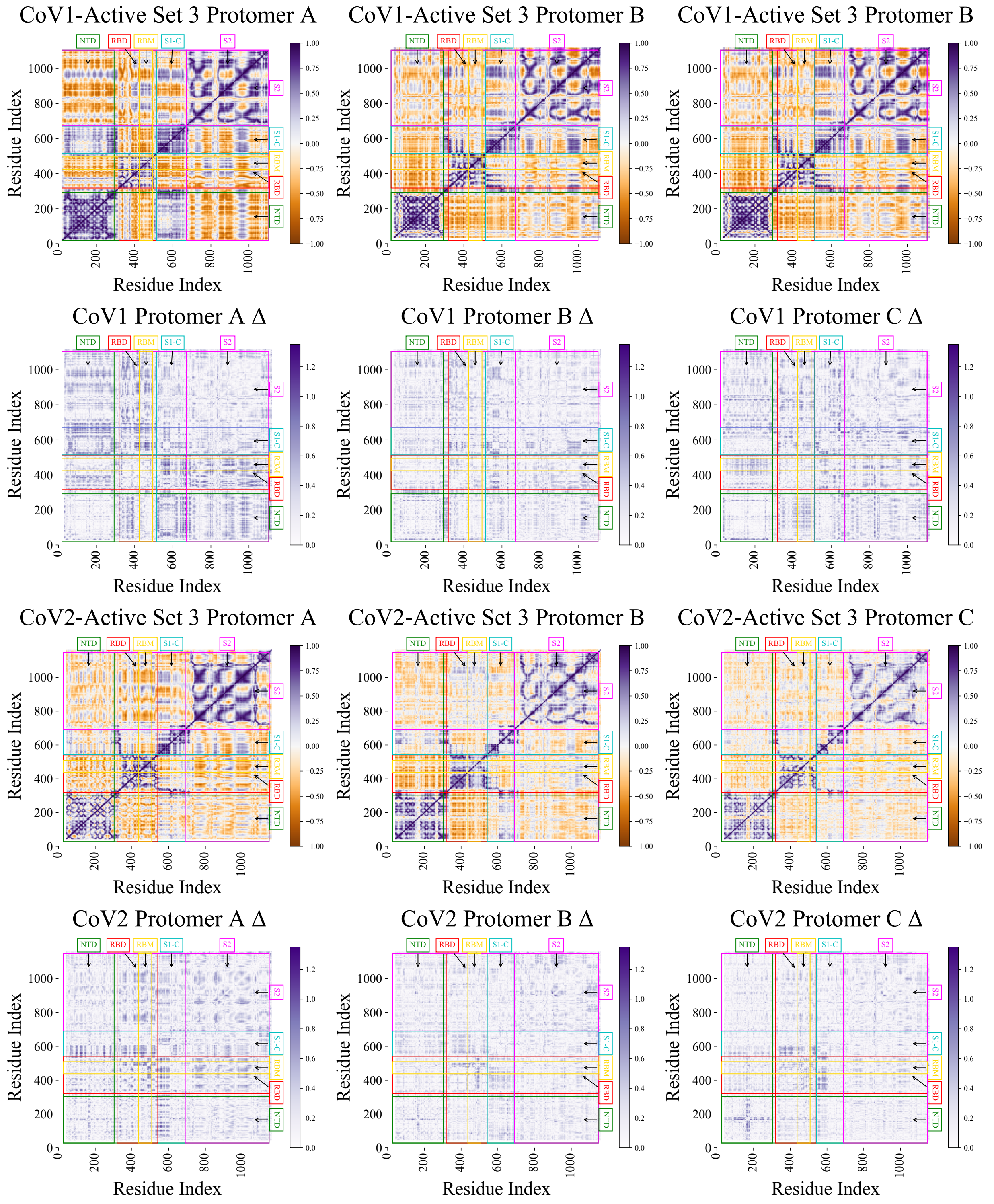

Figure S10: DNA correlation heat maps and Δ matrix for all protomers from Set 3 of the CoV-1 and CoV-2 active spike simulations. (A) DNA heat maps showing the correlation of motions for the CoV-1 active (Set 3) protomers (first row) and the difference matrices (second row). The reference matrix from Figure S7 was used for Δ matrix calculations. **(B)** DNA heat maps showing the correlation of motions for the CoV-2 active (Set 3) protomers (third row) and the difference matrices (fourth row). The reference matrix from Figure S8 was used for Δ matrix calculations.

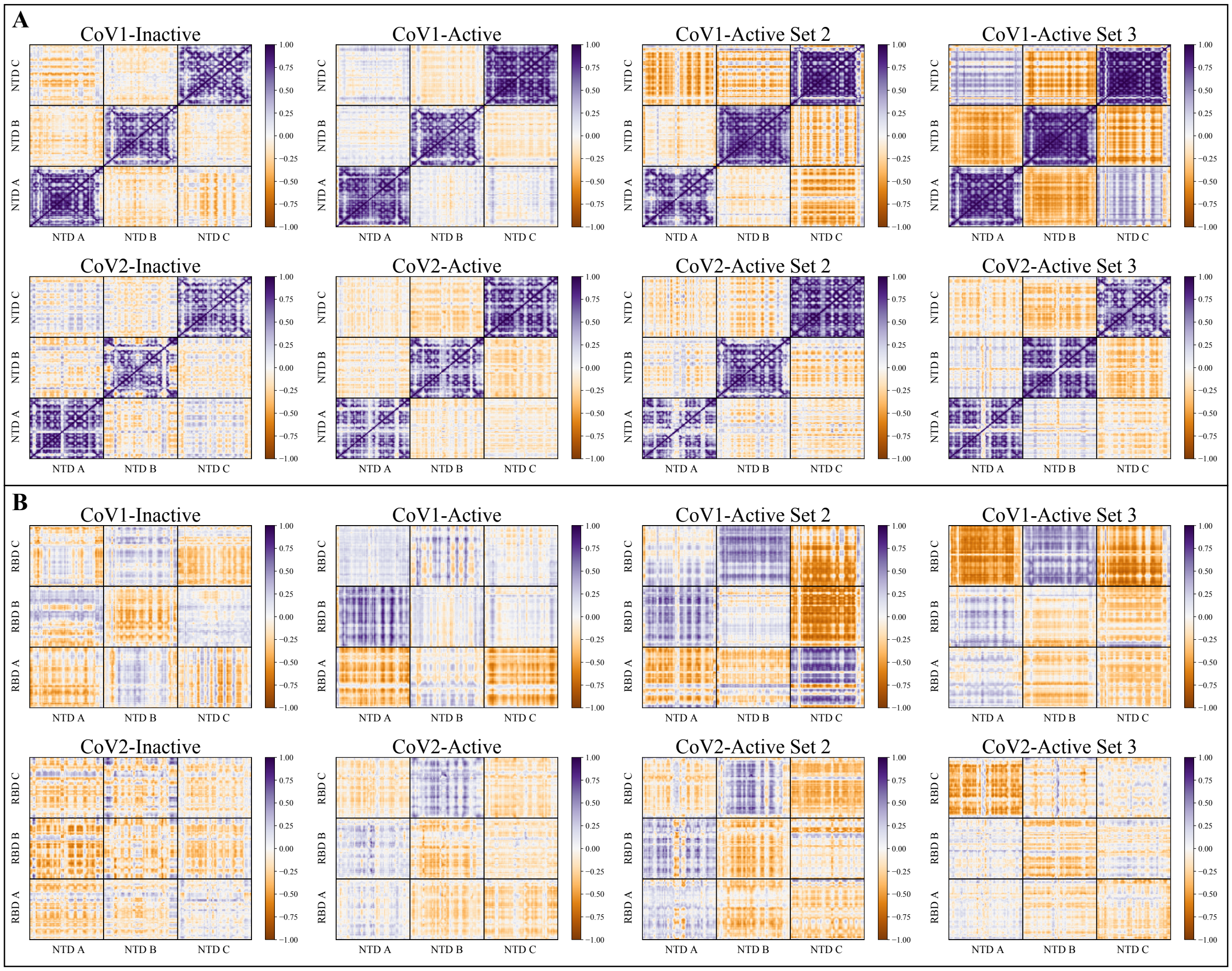

Figure S11: **Dynamic network analysis shows that inter-protomer correlations and anticorrelations are relatively strong in the active CoV-1 spike protein simulations.** **(A)** DNA heat maps showing the correlation of motion between the NTD regions of different protomers. **(B)** DNA heat maps showing the correlation of motion between the NTD and RBD regions of different protomers. Correlations are shown in purple and anti-correlations are shown in orange, with the darker colors indicating greater correlation/anti-correlation.

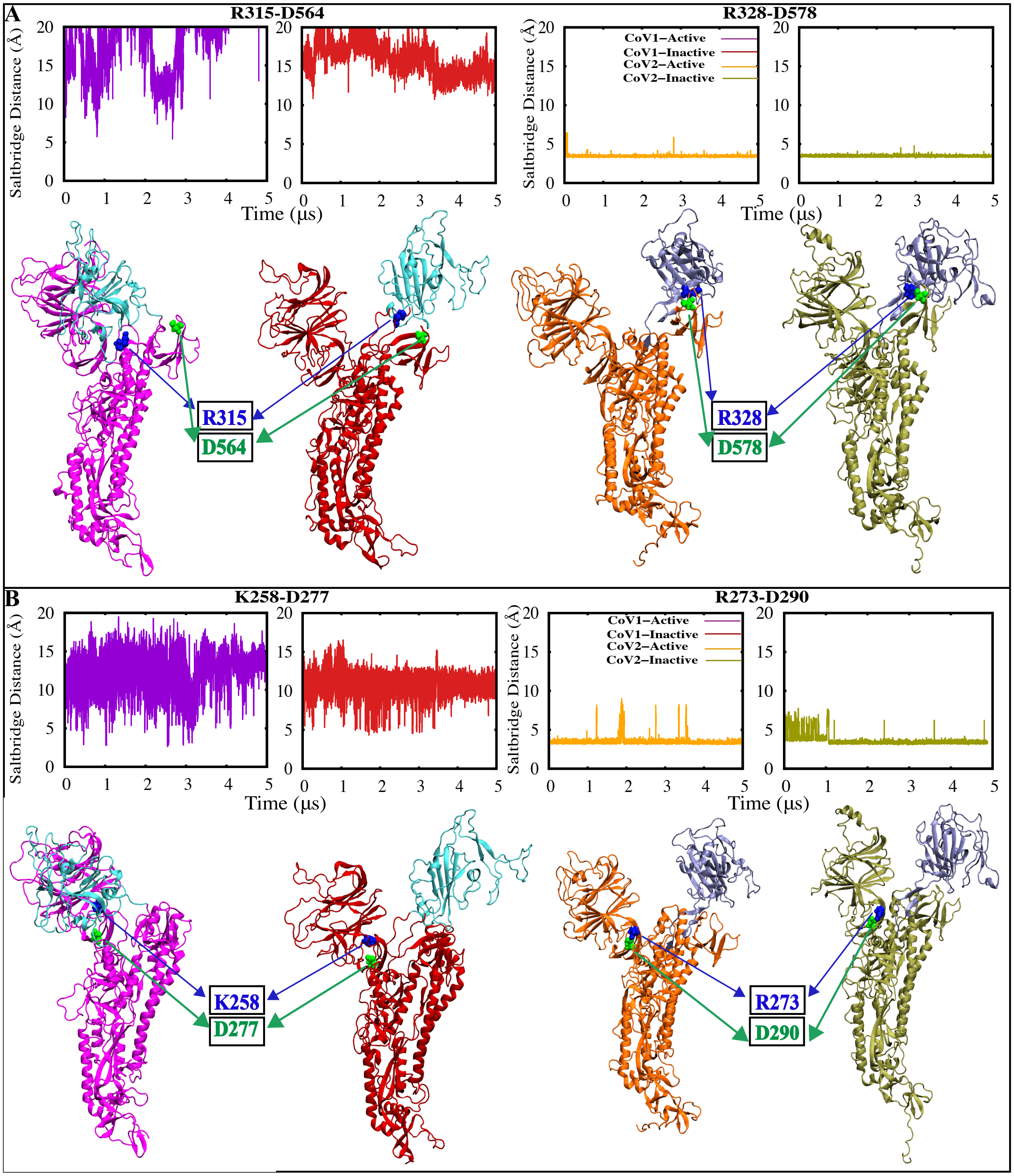

Figure S12: **Conserved residues show distinct differential behavior in the CoV-1 and CoV-2 spike proteins.** Time series and visual representation of the minimum salt-bridge distance for **(A)** R315/328 (blue) - D564/578 (green) and **(B)** K258/R273 (blue) - D277/290 (green), shows that salt-bridges are formed in the CoV-2 spike protein but are absent in the CoV-1 spike protein. These salt-bridges potentially contribute to the higher relative stability of the CoV-2 spike protein. CoV-1 inactive is colored red, CoV-1 active is colored magenta, CoV-2 inactive is colored olive-green and CoV-2 active is colored orange.

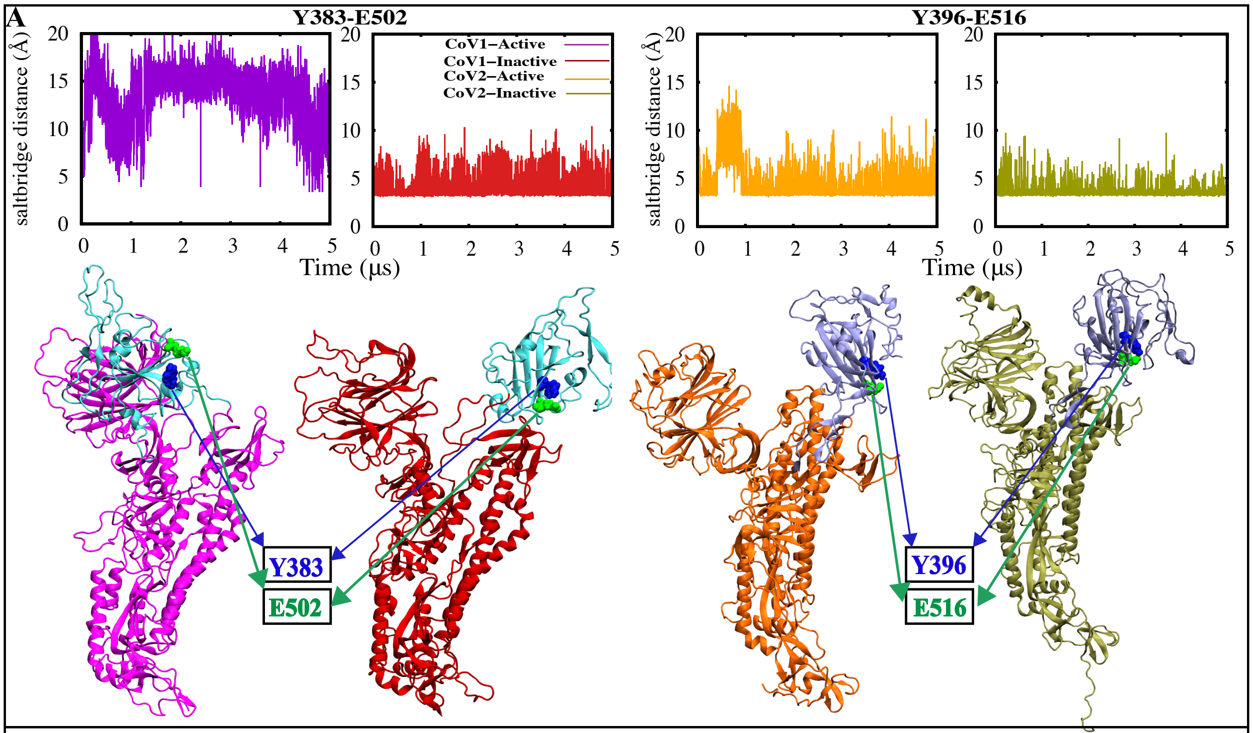

B

R315-D564	Occupancy (%)/Protomer A	B	C
CoV1-active (set 1)	0	0	0
CoV1-active (set 2)	0	0	0
CoV1-active (set 3)	0	0	0
CoV1-inactive	0	0	0
**R328-D578**			
CoV2-active (set 1)	100	76	0.31
CoV2-active (set 2)	59	11	0.95
CoV2-active (set 3)	98	5.2	0.16
CoV2-inactive	100	100	100

K258-D277	Occupancy (%)/Protomer A	B	C
CoV1-active (set 1)	0.96	2.4	1.9
CoV1-active (set 2)	0.34	0.12	0.45
CoV1-active (set 3)	0.09	0.34	0.61
CoV1-inactive	0.38	2.02	20
**R273-D290**			
CoV2-active (set 1)	98	64	94
CoV2-active (set 2)	100	98	85
CoV2-active (set 3)	100	96	96
CoV2-inactive	94	95	87

Y383-E502	Occupancy (%)/Protomer A	B	C
CoV1-active (set 1)	0.02	29	55
CoV1-active (set 2)	0.01	26	62
CoV1-active (set 3)	0.09	31	44
CoV1-inactive	64	21	98
**Y396-E516**			
CoV2-active (set 1)	65	66	94
CoV2-active (set 2)	27	67	80
CoV2-active (set 3)	31	26	45
CoV2-inactive	14	45	1.2

Figure S13: Hydrogen bond analysis for a conserved residue pair within the RBD. (A) Time series and visual representation of the minimum H-bond donor-acceptor distance between Y383/396 (blue) and E502/516 (green), in the CoV-1 and CoV-2 spike respectively. CoV-1 inactive is colored red, CoV-1 active is colored magenta, CoV-2 inactive is colored olive green and CoV-2 active is colored orange. Table **(B)** shows the occupancy (%) of the salt-bridge and hydrogen-bond interactions between conserved residue pairs, for all protomers from all simulation sets.

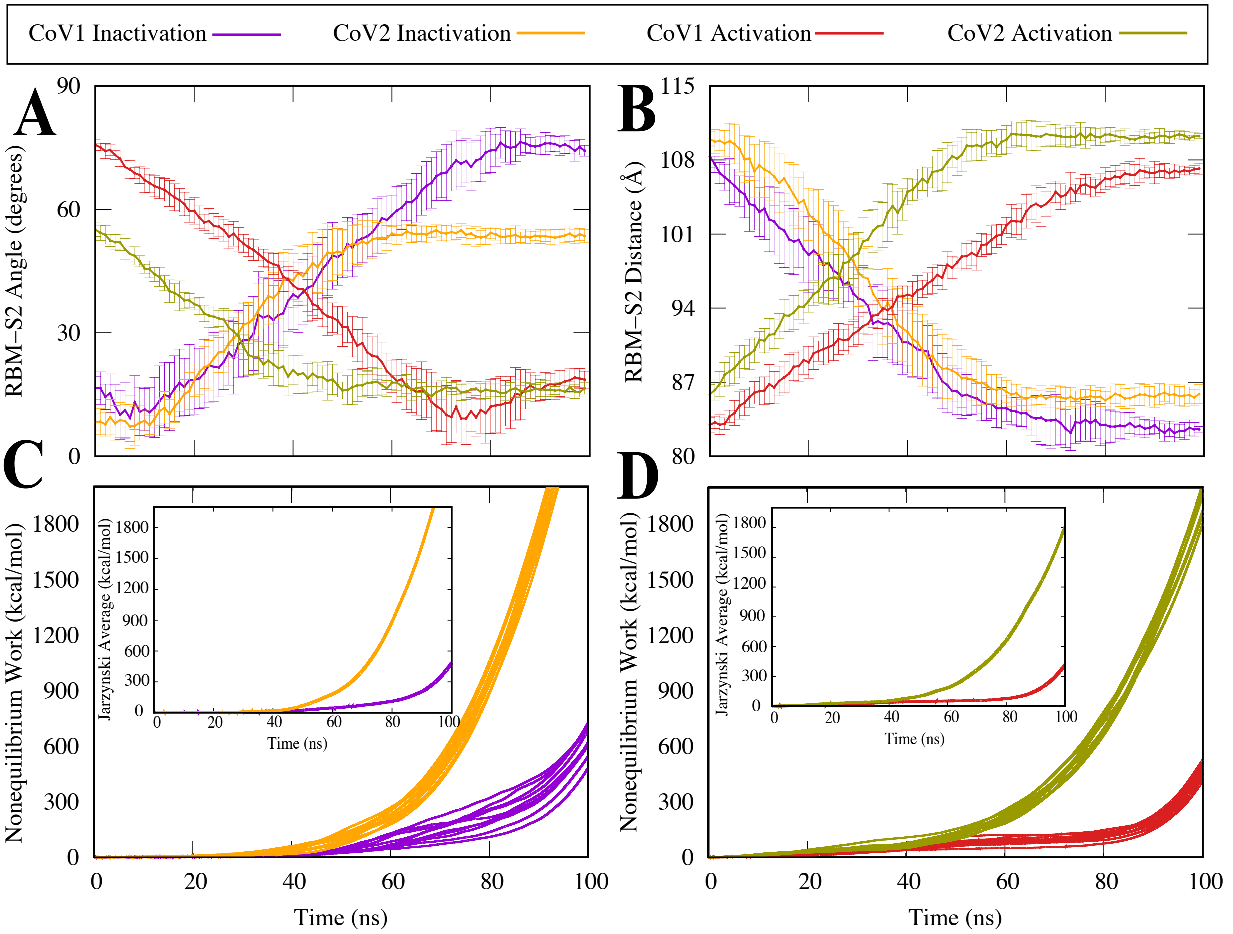

Figure S14: **Three protomer SMD simulations.** **(A)** RBM-S2 Angle between the beta sheet region of the RBM and the alpha helical region of S2, shown as a function of time. Protomer activation is characterized by a decrease in the RBM-S2 angle. **(B)** RBM-S2 COM Distance between the beta sheet region of the RBM and the alpha helical region of S2, shown as shown as a function of time. Protomer activation is characterized by an increase in the RBM-S2 distance.**(C,D)** Accumulated non-equilibrium work as a function of simulation time. Inset: Jarzynski average of non-equilibrium work.

Supporting Movie : Comparative visualization of the conformational dynamics of the active CoV-1 and CoV-2 spike proteins. The movie shows the 5-microsecond long simulations of initially active SARS CoV-1 (right) and 2 (left) spike proteins (Simulation Set 1), where the CoV-1 spike transitions to a pseudo-inactive state, where the RBD and NTD interact, while CoV-2 spike stays active. The proteins are shown in cartoon representation with a color code similar to Fig. 1A,B.

References

(1) Walls, A. C.; Park, Y.-J.; Tortorici, M. A.; Wall, A.; McGuire, A. T.; Veesler, D. Structure, Function, and Antigenicity of the SARS-CoV-2 Spike Glycoprotein. *Cell* **2020**, *181*, 281 – 292.e6.

(2) Yuan, Y.; Cao, D.; Zhang, Y.; Ma, J.; Qi, J.; Wang, Q.; Lu, G.; Wu, Y.; Yan, J.; Shi, Y. et al. Cryo-EM structures of MERS-CoV and SARS-CoV spike glycoproteins reveal the dynamic receptor binding domains. *Nature Communications* **2017**, *8*, 15092.

(3) Eswar, N.; Eramian, D.; Webb, B.; Shen, M. Y.; Sali, A. *Kobe B., Guss M., Huber T. (eds) Structural Proteomics. Methods in Molecular Biology*; Humana Press, 2008; Vol. 426; pp 145–159.

(4) Jo, S.; Kim, T.; Iyer, V. G.; Im, W. CHARMM-GUI: A Web-Based Graphical User Interface for CHARMM. *J. Comp. Chem.* **2008**, *29*, 1859–1865.

(5) Lee, J.; Cheng, X.; Swails, J. M.; Yeom, M. S.; Eastman, P. K.; Lemkul, J. A.; Wei, S.; Buckner, J.; Jeong, J. C.; Qi, Y. et al. CHARMM-GUI input generator for NAMD, GROMACS, AMBER, OpenMM, and CHARMM/OpenMM simulations using the CHARMM36 additive force field. *J. Chem. Theory Comp.* **2016**, *12*, 405–413.

(6) Phillips, J. C.; Braun, R.; Wang, W.; Gumbart, J.; Tajkhorshid, E.; Villa, E.; Chipot, C.; Skeel, R. D.; Kale, L.; Schulten, K. Scalable molecular dynamics with NAMD. *J. Comp. Chem.* **2005**, *26*, 1781–1802.

(7) Huang, J.; Rauscher, S.; Nawrocki, G.; Ran, T.; Feig, M.; De Groot, B. L.; Grubmüller, H.; MacKerell, A. D. CHARMM36m: An improved force field for folded and intrinsically disordered proteins. *Nature Methods* **2017**, *14*, 71–73.

(8) Reid, J. K. In *Large Sparse Sets of Linear Equations*; Reid, J. K., Ed.; Academic Press: London, 1971; pp 231–254.

(9) Martyna, G. J.; Tobias, D. J.; Klein, M. L. Constant Pressure Molecular Dynamics Algorithms. *J. Chem. Phys.* **1994**, *101*, 4177–4189.

(10) Darden, T.; York, D.; Pedersen, L. Particle Mesh Ewald: An $N \cdot \log(N)$ Method for Ewald Sums in Large Systems. *J. Chem. Phys.* **1993**, *98*, 10089–10092.

(11) Shaw, D. E.; Grossman, J.; Bank, J. A.; Batson, B.; Butts, J. A.; Chao, J. C.; Deneroff, M. M.; Dror, R. O.; Even, A.; Fenton, C. H. et al. Anton 2: raising the bar for performance and programmability in a special-purpose molecular dynamics supercomputer. Proceedings of the International Conference for High Performance Computing, Networking, Storage and Analysis. 2014; pp 41–53.

(12) Humphrey, W.; Dalke, A.; Schulten, K. VMD: Visual molecular dynamics. *Journal of Molecular Graphics & Modelling* **1996**, *14*, 33–38.

(13) Amadei, A.; Linnsen, A. B. M.; Berendsen, H. J. C. Essential Dynamics of Proteins. *Proteins: Struct., Func., Gen.* **1993**, *17*, 412–425.

(14) Bakan, A.; Meireles, L. M.; Bahar, I. ProDy: Protein Dynamics Inferred from Theory and Experiments. *Bioinformatics* **2011**, *27*, 1575–1577.

(15) Sethi, A.; Eargle, J.; Black, A. A.; Luthey-Schulten, Z. Dynamical networks in tRNA:protein complexes. *Proc. Natl. Acad. Sci. USA* **2009**, *106*, 6620–6625.

(16) Brown, D. K.; Penkler, D. L.; Amamuddy, O. S.; Ross, C.; Atilgan, A. R.; Atilgan, C.; Bishop, Ö. T. MD-TASK: a software suite for analyzing molecular dynamics trajectories. *Bioinformatics* **2017**, *33*, 2768 – 2771.

(17) Jarzynski, C. Nonequilibrium Equality for Free Energy Differences. *Phys. Rev. Lett.* **1997**, *78*, 2690–2693.

(18) Moradi, M.; Sagui, C.; Roland, C. Calculating relative transition rates with driven nonequilibrium simulations. *Chem. Phys. Lett.* **2011**, *518*, 109–113.

(19) Moradi, M.; Tajkhorshid, E. Mechanistic Picture for Conformational Transition of a Membrane Transporter at Atomic Resolution. *Proc. Natl. Acad. Sci. USA* **2013**, *110*, 18916–18921.

(20) Moradi, M.; Sagui, C.; Roland, C. Invstigating rare events with nonequilibrium work measurements: I. nonequilibrium transition path probabilities. *J. Chem. Phys.* **2014**, *140*, 034114.

(21) Moradi, M.; Sagui, C.; Roland, C. Invstigating rare events with nonequilibrium work measurements: II. transition and reaction rates. *J. Chem. Phys.* **2014**, *140*, 034115.
